# DNA Methylation Epigenetically Regulates Gene Expression in Burkholderia cenocepacia and Controls Biofilm Formation, Cell Aggregation, and Motility

**DOI:** 10.1128/mSphere.00455-20

**Published:** 2020-07-15

**Authors:** Ian Vandenbussche, Andrea Sass, Marta Pinto-Carbó, Olga Mannweiler, Leo Eberl, Tom Coenye

**Affiliations:** a Laboratory of Pharmaceutical Microbiology, Ghent University, Ghent, Belgium; b Department of Plant and Microbial Microbiology, University of Zurich, Zurich, Switzerland; University of Iowa

**Keywords:** *Burkholderia*, DNA methylation, epigenetics

## Abstract

Respiratory tract infections by the opportunistic pathogen Burkholderia cenocepacia often lead to severe lung damage in cystic fibrosis (CF) patients. New insights in how to tackle these infections might emerge from the field of epigenetics, as DNA methylation is an important regulator of gene expression. The present study focused on two DNA methyltransferases (MTases) in B. cenocepacia strains J2315 and K56-2 and their role in regulating gene expression. *In silico* predicted DNA MTase genes BCAL3494 and BCAM0992 were deleted in both strains, and the phenotypes of the resulting deletion mutants were studied: deletion mutant ΔBCAL3494 showed changes in biofilm structure and cell aggregation, while ΔBCAM0992 was less motile. B. cenocepacia wild-type cultures treated with sinefungin, a known DNA MTase inhibitor, exhibited the same phenotype as DNA MTase deletion mutants. Single-molecule real-time sequencing was used to characterize the methylome of B. cenocepacia, including methylation at the origin of replication, and motifs CACAG and GTWWAC were identified as targets of BCAL3494 and BCAM0992, respectively. All genes with methylated motifs in their putative promoter region were identified, and qPCR experiments showed an upregulation of several genes, including biofilm- and motility-related genes, in MTase deletion mutants with unmethylated motifs, explaining the observed phenotypes in these mutants. In summary, our data confirm that DNA methylation plays an important role in regulating the expression of B. cenocepacia genes involved in biofilm formation, cell aggregation, and motility.

**IMPORTANCE** CF patients diagnosed with Burkholderia cenocepacia infections often experience rapid deterioration of lung function, known as cepacia syndrome. B. cenocepacia has a large multireplicon genome, and much remains to be learned about regulation of gene expression in this organism. From studies in other (model) organisms, it is known that epigenetic changes through DNA methylation play an important role in this regulation. The identification of B. cenocepacia genes of which the expression is regulated by DNA methylation and identification of the regulatory systems involved in this methylation are likely to advance the biological understanding of B. cenocepacia cell adaptation via epigenetic regulation. In time, this might lead to novel approaches to tackle B. cenocepacia infections in CF patients.

## Abstract (precis)

CF patients diagnosed with Burkholderia cenocepacia infections often experience rapid deterioration of lung function, known as cepacia syndrome. B. cenocepacia has a large multireplicon genome, and much remains to be learned about regulation of gene expression in this organism. From studies in other (model) organisms, it is known that epigenetic changes through DNA methylation play an important role in this regulation. The identification of B. cenocepacia genes of which the expression is regulated by DNA methylation and identification of the regulatory systems involved in this methylation are likely to advance the biological understanding of B. cenocepacia cell adaptation via epigenetic regulation. In time, this might lead to novel approaches to tackle B. cenocepacia infections in CF patients.

## INTRODUCTION

Burkholderia cenocepacia, a member of the Burkholderia cepacia complex (Bcc), is an aerobic Gram-negative bacterium that can be isolated from soil and water (1–3). B. cenocepacia is also known as an opportunistic pathogen in immunocompromised patients (4–6). Infection of the upper airways in cystic fibrosis (CF) patients often leads to severe illness, typically referred to as cepacia syndrome (1, 7). CF patients diagnosed with cepacia syndrome experience a progressive decrease in lung function, often accompanied by bacteremia and sepsis. If left untreated, cepacia syndrome can lead to death within weeks (8, 9). The genome of B. cenocepacia is complex (with usually three large replicons), with a high GC content (67%) and large size, comprising approximately 8.06 Mb (10). The species has been classified into different phylogenetic clusters and subdivided into lineages, including the highly transmissible ET-12 lineage that harbors B. cenocepacia strains J2315 and K56-2 (11, 12).

Epigenetics is the study of heritable changes in gene expression without changes in the actual genomic sequence. In bacterial genomes, epigenetic control is exerted by DNA methyltransferase enzymes (MTases) (13–15). DNA MTases originate from restriction-modification (RM) systems, early defense mechanisms in bacteria with an active interplay between endonucleases and DNA MTases, which cleave foreign DNA but protect the organism’s own genome. In addition, discovery of orphan DNA MTases, enzymes without a cognate endonuclease, shows that DNA MTases are not exclusively dependent on the presence of the restriction part to function as regulator of gene expression (16).

DNA MTases interact with specific DNA recognition sites and transfer a CH_3_ group from a methyl donor, mostly *S*-adenosylmethionine (SAM), to a cytosine (*C*_5_-methyl cytosine or *N*_4_-methyl cytosine) or adenine (*N*_6_-methyl adenine) base (17, 18). As methylated bases change the binding affinity of DNA binding proteins, methylation at regulatory regions allows bacteria to regulate gene expression at the level of transcription (19, 20). While both cytosine and adenine methylation occur in eukaryotic and prokaryotic cells, C_5_-methyl cytosine is the archetypal eukaryotic base methylation signature (16, 21). Conversely, in prokaryotes, *N*_6_-methyl adenine is the most important base modification involved in gene expression regulation (22). In addition to this, studies with DNA MTases *Dam* (deoxyadenosine methyltransferase) and *Dcm* (DNA cytosine MTase) in Escherichia coli have demonstrated that, besides having a regulatory function, DNA MTases also take part in crucial cellular processes like DNA replication initiation or methyl-directed mismatch repair (21, 23).

Detection of (genome-wide) DNA methylation patterns has been challenging in the past. The use of specific restriction enzymes with affinity for methylated sites, followed by a comparison of the resulting fragment lengths, gave a good impression of methylation of the treated DNA at one particular area, but global methylation analysis was until recently difficult at best (21, 24). The rise of next-generation sequencing and single-molecule real-time (SMRT) technologies tremendously improved the quality of methylome analyses, but it also made it much more accessible (25, 26). SMRT sequencing uses a sequencing-by-synthesis approach with fluorescently labeled nucleotides. Pulse width, the signal of nucleotide incorporation, and interpulse duration, the time between two incorporations, allow discrimination between incorporated bases and their methylation status (27).

The purpose of the present study is to understand how DNA methylation regulates gene expression in B. cenocepacia. To this end, a genome-wide methylome analysis was carried out, and genes under DNA methylation regulation were identified.

## RESULTS

### Identification of B. cenocepacia DNA MTases.

All predicted DNA MTase genes in the B. cenocepacia J2315 genome were identified using REBASE (see Table S1 in the supplemental material). DNA MTase genes BCAL3494 and BCAM0992, widely distributed within the genus *Burkholderia*, were selected for further analysis. Gene BCAL3494, located on the first replicon of B. cenocepacia, is a type III methyltransferase that is part of an RM system, together with a restriction enzyme encoded by the neighboring gene BCAL3493. Gene BCAM0992 is located on the second replicon and apparently does not have any adjacent genes coding for restriction enzymes. Instead, BCAM0992 is part of the *trp* operon that comprises genes involved in tryptophan biosynthesis and is located between *trpB* (BCAM0991) and *trpA* (BCAM0993). It is potentially under transcriptional control of proteins regulating this operon, including the *trp* repressor as described in E. coli (28). The presence of MTase genes in *trp-*containing operons has been reported before, as in most enterobacteria the gene of the adenine-specific MTase Dam is located close to *trpS* (29). Although BCAM0992 was identified as an orphan MTase gene, it strongly resembles MTase genes of type II RM systems, in which the restriction and modification enzymes act separately and are not dependent on each other (30). To investigate the influence of BCAL3494 and BCAM0992 on bacterial physiology, deletion mutants were constructed (Fig. S1). For the other DNA MTase genes in B. cenocepacia J2315 identified with REBASE (Table S1), homologues in different *Burkholderia* strains could not be found; these genes were not further investigated in the present study.

10.1128/mSphere.00455-20.1 FIG S1 Genome context of deleted DNA MTase genes BCAL3494 (top) and BCAM0992 (bottom). The red arrows indicate the boundaries of the deleted part. For gene BCAL3494, adjacent restriction gene BCAL3493 and hypothetical genes BCAL3488 to BCAL3492 were deleted as well. Download FIG S1, TIF file, 0.3 MB. Copyright © 2020 Vandenbussche et al. 2020 Vandenbussche et al. This content is distributed under the terms of the Creative Commons Attribution 4.0 International license.

10.1128/mSphere.00455-20.7 TABLE S1 All DNA MTase genes in the B. cenocepacia J2315 genome identified by REBASE (* BLASTn search against the genus *Burkholderia*, default screening parameters were used) and the methylation motifs detected by SMRT sequencing. Most of the MTase genes are predominantly expressed in the exponential growth phase. Methylated bases in bold and underlined; 6mA, *N*_6_-methyl adenine; 4mC, *N*_4_-methyl cytosine; F, forward strand; R, reverse strand. *, wild-type strain J2315 percentages. Download Table S1, DOCX file, 0.02 MB. Copyright © 2020 Vandenbussche et al. 2020 Vandenbussche et al. This content is distributed under the terms of the Creative Commons Attribution 4.0 International license.

### Phenotype of mutant strains.

**FIG 1:**
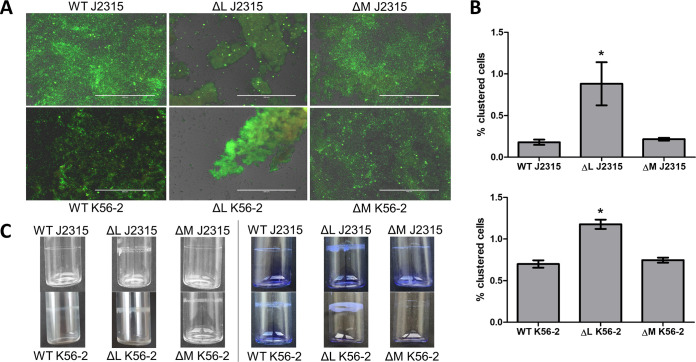
Effect of DNA MTase deletion on biofilm structure, cell aggregation, and pellicle formation in B. cenocepacia J2315 and K56-2. (A) Microscopic images of LIVE/DEAD-stained biofilms, grown in microtiter plate wells for 24 h. White bar (200 μm) for scale. (B) Clustering of cells in planktonic cultures, quantified with flow cytometry. (C) Pellicle formation inside glass tubes after 24 h of static incubation. Left pictures represent unstained samples, and right pictures display pellicles stained with crystal violet. *n* = 3; *, *P* < 0.05 compared to wild type; error bars represent the standard error of the mean (SEM). WT, wild type; ΔL, deletion mutant ΔBCAL3494; ΔM, deletion mutant ΔBCAM0992.

MTase genes BCAL3494 (including neighboring genes BCAL3488 to -3492) and BCAM0992 were deleted in B. cenocepacia strains J2315 and K56-2 (Fig. S1), and the phenotype of the deletion mutants was investigated in detail. No differences in growth rate during exponential phase were observed between wild-type and mutant strains when cultured in phosphate-buffered mineral medium (Fig. S2). Microscopic analysis showed a different, more clustered biofilm morphology for both BCAL3494 deletion mutants (ΔBCAL3494) compared to wild-type strains, whereas the biofilm structure of the BCAM0992 deletion mutants (ΔBCAM0992) did not differ from wild type (Fig. 1A). Cell aggregation in planktonic cultures was investigated using flow cytometry (Fig. 1B). The degree of aggregation in the BCAL3494 mutant strains was significantly higher (*P* value J2315, 0.049; *P* value K56-2, 0.001) than in the corresponding wild-type strains. Also, the ability to form a pellicle, a biofilm-like structure at the air-liquid interface, was investigated (Fig. 1C). Pellicle formation was increased for both ΔBCAL3494 mutants compared to wild-type strains and to ΔBCAM0992 mutants. Complemented mutant strains *c*ΔBCAL3494 and *c*ΔBCAM0992 did not differ significantly from wild type in these experiments, as shown for strain J2315 in Fig. S3.

10.1128/mSphere.00455-20.2 FIG S2 Growth of B. cenocepacia J2315 (left) and K56-2 (right) in phosphate-buffered minimal medium. WT, wild type; ΔL, deletion mutant ΔBCAL3494; ΔM, deletion mutant ΔBCAM0992. Insets show same curves but with *y* axis in log scale. Download FIG S2, TIF file, 0.4 MB. Copyright © 2020 Vandenbussche et al. 2020 Vandenbussche et al. This content is distributed under the terms of the Creative Commons Attribution 4.0 International license.

10.1128/mSphere.00455-20.3 FIG S3 Biofilm formation, cell aggregation, and pellicle formation of complemented DNA MTase mutants in B. cenocepacia J2315. (A) Microscopic images of LIVE/DEAD-stained biofilms, grown in microtiter plate wells for 24 h. White bar (200 μm) for scale. (B) Clustering of cells in planktonic cultures, analyzed with flow cytometry. (C) Pellicle formation inside glass tubes after 24 h of static incubation, stained with crystal violet. *n* = 3; error bars represent the standard error of the mean (SEM). WT, wild type; ΔL pJH2 and ΔM pJH2, mutant strains with empty vector pJH2 (vector control); *c*ΔL pJH2 and *c*ΔM pJH2, deletion mutants complemented with genes BCAL3494 and BCAM0992. Download FIG S3, TIF file, 1.7 MB. Copyright © 2020 Vandenbussche et al. 2020 Vandenbussche et al. This content is distributed under the terms of the Creative Commons Attribution 4.0 International license.

**FIG 2:**
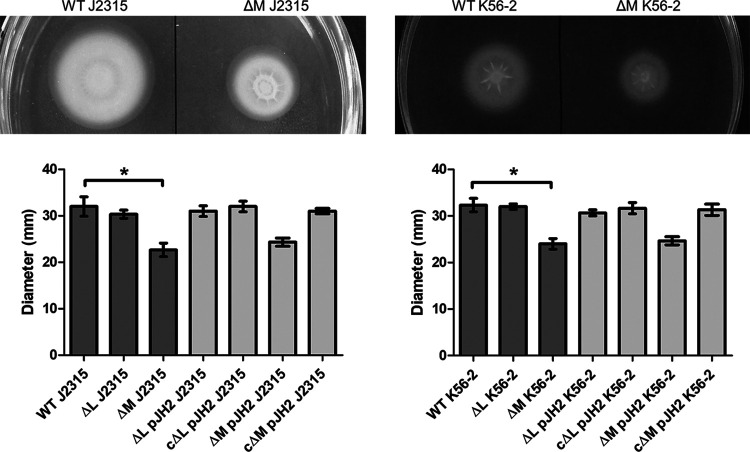
Swimming motility of DNA MTase deletion mutants. Diameters were measured after 24 h (K56-2) or 32 h (J2315). *n* = 3; *, *P* < 0.05 compared to wild type, error bars represent the SEM. WT, wild type; ΔL, deletion mutant ΔBCAL3494; ΔM, deletion mutant ΔBCAM0992; ΔL pJH2 and ΔM pJH2, mutant strains with empty vector pJH2 (vector control); *c*ΔL pJH2 and *c*ΔM pJH2, deletion mutants complemented with genes BCAL3494 and BCAM0992.

Motility of all strains was assessed using a swimming motility assay on 0.3% agar plates. After 24 h (strain K56-2) and 32 h (strain J2315), plates were photographed and diameters were measured (Fig. 2). Diameters were significantly smaller for both ΔBCAM0992 mutants compared to wild type (*P* value J2315, 0.002; *P* value K56-2, <0.001). Both ΔBCAL3494 mutants, as well as the complemented mutants, were identical to wild type. We also investigated swarming motility, but no significant differences between the different strains were observed.

Galleria mellonella (wax moth) larvae were used as infection model to assess the virulence of B. cenocepacia J2315 in the absence of methylation. After both 24 h and 48 h, the percentage of survival in the ΔBCAL3494 mutant group was higher than in the other groups. However, the observed difference was not statistically significant (*P* value, 0.321). After 72 h, all larvae were reported dead (Fig. S4). Although DNA methylation depletion did not lead to a decreased virulence in G. mellonella, the role of adaptive immunity found in higher organisms and the behavior of immune cells toward such MTase mutants with altered biofilm production or swimming motility remains to be investigated.

10.1128/mSphere.00455-20.4 FIG S4 Virulence of B. cenocepacia J2315 in the G. mellonella infection model (*n* = 8 per group). At time points 24 h and 48 h, the group infected with deletion mutant ΔBCAL3494 showed a higher percentage of survival than the other groups. PS, physiological saline (control group); c Δ strains, complemented mutant strains; WT, wild type. Download FIG S4, TIF file, 0.4 MB. Copyright © 2020 Vandenbussche et al. 2020 Vandenbussche et al. This content is distributed under the terms of the Creative Commons Attribution 4.0 International license.

### Effect of the DNA MTase inhibitor sinefungin on methylation-dependent phenotypes.

**FIG 3:**
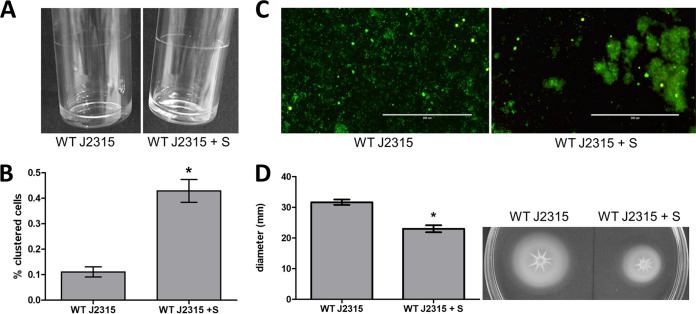
Effect of DNA MTase inhibitor sinefungin on biofilm and pellicle formation, cell aggregation, and motility. (A) Pellicle formation inside glass tubes after 24 h of static incubation. (B) Clustering of planktonic cultures analyzed with flow cytometry. (C) Microscopic images of LIVE/DEAD-stained biofilms, grown on plastic surfaces in microtiter plates for 24 h. (D) Swimming motility of treated and untreated samples. *n* = 3; *, *P* < 0.05 compared to wild type; error bars represent the SEM. WT, wild type; +S, medium supplemented with 50 μg/ml sinefungin.

Sinefungin, a structural analog of SAM and known for blocking base methylation in other bacteria such as Streptococcus pneumoniae (31), was used as a DNA MTase inhibitor. The MIC of sinefungin in B. cenocepacia J2315 and K56-2 was determined and was higher than 200 μg/ml. Both strains were exposed to sinefungin concentrations below the MIC of sinefungin (50 μg/ml) to ensure that any effect observed was not due to growth inhibition by sinefungin, and the effect on biofilm formation, pellicle formation, cell aggregation, and motility was quantified (Fig. 3). Bacteria exposed to sinefungin produced more pellicle mass, showed a higher degree of cell aggregation (*P* value, 0.003), had a different biofilm morphology, and were less motile (*P* value, 0.004). These findings suggest that chemically blocking DNA methylation or deleting genes responsible for DNA methylation leads to the same phenotypes in B. cenocepacia J2315 and K56-2.

### Methylome analysis.

**FIG 4:**
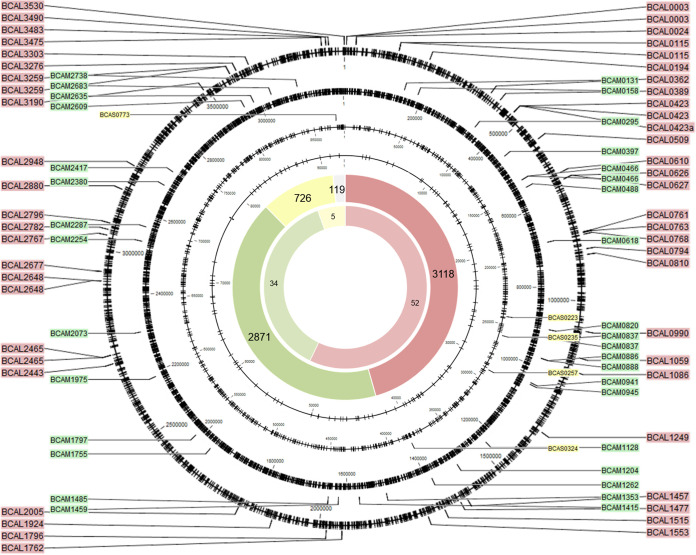
Genomic position of all methylated CACAG motifs. Black circles represent the four replicons of B. cenocepacia; black ticks mark the motif locations. The total number of methylated CACAG motifs and methylated CACAG motifs in promoter regions, per replicon (red, replicon 1; green, replicon 2; yellow, replicon 3; gray, plasmid), is shown on the large and small inner circle, respectively. The positions and names of genes with methylated promoter regions are indicated with colored labels (same color code).

**FIG 5:**
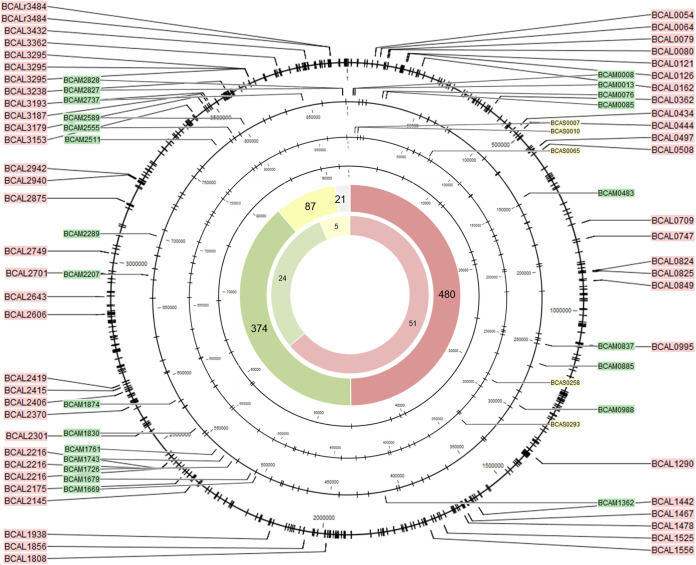
Genomic position of all methylated GTWWAC motifs. Black circles represent the four replicons of B. cenocepacia; black ticks mark the motif locations. The total number of methylated GTWWAC motifs and methylated GTWWAC motifs in promoter regions, per replicon (red, replicon 1; green, replicon 2; yellow, replicon 3; gray, plasmid), is shown on the large and small inner circle, respectively. The positions and names of genes with methylated promoter regions are indicated with colored labels (same color code).

Using SMRT sequencing (PacBio), the complete methylome of B. cenocepacia J2315 and K56-2 was identified. Only data for strain J2315 are reported below, as data for strain K56-2 were highly comparable (Fig. 4 and 5). Two distinct methylated motifs were identified in the wild-type strain: CACAG and GTWWAC. The CACAG motif was methylated at the fourth position on the forward strand, whereas the GTWWAC motif was methylated at the fifth position on both the forward and reverse strands. Although all CACAG and GTWWAC motifs were methylated in the wild-type strains, methylation of the CACAG motif was absent in the ΔBCAL3494 deletion mutants, and likewise, no methylation of the GTWWAC motif was seen in the ΔBCAM0992 mutants (Table S1). This demonstrates that MTase BCAL3494 recognizes the CACAG motif, while MTase BCAM0992 recognizes the GTWWAC motif.

The location of every methylated CACAG and GTWWAC motif was mapped (Fig. 4 and 5). In all, 6,834 methylated CACAG motifs and 961 methylated GTWWAC motifs were found, of which the majority was present on the first replicon (CACAG, 45.6%; GTWWAC, 49.9%), followed by the second replicon (CACAG, 42.1%; GTWWAC, 38.9%), the third replicon (CACAG, 10.6%; GTWWAC, 9.0%), and the plasmid (CACAG, 1.7%; GTWWAC, 2.2%). Subsequently, all genes with methylated motifs in their promoter region, here defined as 60 bases upstream of the transcription start site, were identified. Ninety-one promoter regions contained methylated CACAG motifs, and 80 promoter regions contained methylated GTWWAC motifs, with most of the motifs being present on the first replicon (Fig. 4 and 5). To reveal possible sites of epigenetic regulation, at which DNA methylation is hindered by binding of a regulator, positions of the few unmethylated motifs were analyzed. However, none of these motifs were found in promoter regions. Functional classes of genes found in the data set of genes with methylated promoters include genes involved in intermediary metabolism, regulation, and transport (Table S2).

10.1128/mSphere.00455-20.8 TABLE S2 Genes with methylated promoter region (CACAG motif and GTWWAC) in J2315 (methylated promoter regions in K56-2 are indicated with +, and nonmethylated promoter regions are indicated with −). Download Table S2, DOCX file, 0.03 MB. Copyright © 2020 Vandenbussche et al. 2020 Vandenbussche et al. This content is distributed under the terms of the Creative Commons Attribution 4.0 International license.

Virtual Footprint was used to elucidate to which transcription factor (TF) binding sites the discovered methylation motifs CACAG and GTWWAC showed any similarity. Data output of the analysis is listed in Table S3. Sequences that contain methylation motif CACAG were similar to the binding site of E. coli K-12 GlpR, while GTWWAC-containing sequences were similar to binding sites of several other E. coli K-12 TFs, including ArcA, Fis, OxyR, and Fur. Interestingly, the latter two have previously been described as TFs with DNA methylation-blocking ability in Salmonella enterica and E. coli (32, 33).

10.1128/mSphere.00455-20.9 TABLE S3 List of TFs that bind to methylation motifs CACAG and GTWWAC, predicted by Virtual Footprint. Bold sequences represent methylation motifs. Consensus sequence based on TF binding in E. coli K-12. Download Table S3, DOCX file, 0.01 MB. Copyright © 2020 Vandenbussche et al. 2020 Vandenbussche et al. This content is distributed under the terms of the Creative Commons Attribution 4.0 International license.

### Expression of genes with a methylated promoter.

**FIG 6:**
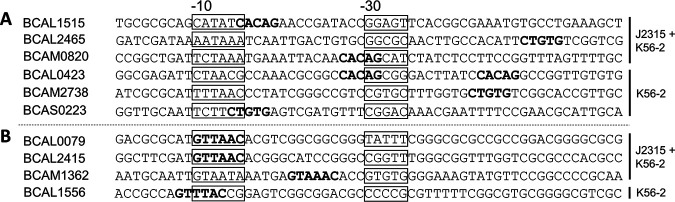
Position of methylated motifs relative to gene start for genes of which the expression is upregulated in DNA MTase deletion mutants. J2315 + K56-2, upregulation in both strains; K56-2, upregulation in strain K56-2 only. (A) Genes with methylated CACAG motifs in their corresponding promoter region. (B) Genes with methylated GTWWAC motifs in their corresponding promoter region. The motifs are marked in bold; the positions of −10 and −30/35 elements in bacterial promoters are framed.

The expression level of genes with methylated promoter regions was determined in wild-type and mutant strains, using qPCR. Expression data for genes with methylated promoter regions are listed in Tables 1 and 2. Volcano plots (Fig. S5) (fold changes plotted against corresponding *P* values) show that most genes tested were upregulated in the mutants compared to the wild-type strains. Six of these genes were significantly upregulated in mutants of both strain backgrounds: BCAL1515, BCAL2465, and BCAM0820 were upregulated in ΔBCAL3494, whereas genes BCAL0079, BCAL2415, and BCAM1362 were upregulated in ΔBCAM0992. Four additional genes were upregulated in K56-2 mutants only: BCAL0423, BCAM2738, and BCAS0223 were upregulated in ΔBCAL3494, and BCAL1556 was upregulated in ΔBCAM0992. Subsequently, the methylated promoter regions of these genes were analyzed in detail (Fig. 6). In most cases, the methylated motif was in close proximity to the −10 or −30/35 element in bacterial promoter regions. As DNA methylation in bacteria can control binding of TFs outside the promoter region (14), we extended the genomic context to look for additional methylation sites upstream of these upregulated genes with a methylated promoter (up to 200 bp), but no additional methylation patterns could be identified.

**TABLE 1:** Expression changes of genes with a methylated CACAG motif in their promoter region in deletion mutants compared to wild type

Locus tag	J2315		K56-2		Gene function
	Fold change	*P* value	Fold change	*P* value	
BCAL0003	0.954	0.791	1.242	0.214	MarR family regulatory protein
BCAL0024	1.477	0.143	0.909	0.678	GidA tRNA uridine 5-carboxymethylaminomethyl modification enzyme
BCAL0423	1.169	0.306	1.948	0.014	DnaA chromosomal replication initiation protein
BCAL0509	1.129	0.473	1.175	0.199	MetK *S*-adenosylmethionine synthetase
BCAL1059	1.129	0.662	0.767	0.457	ArgD bifunctional *N*-succinyldiaminopimelate-aminotransferase/acetylornithine transaminase protein
BCAL1457	1.343	0.309	1.793	0.056	LysR family regulatory protein
BCAL1515	1.790	0.032	1.869	0.012	SucA 2-oxoglutarate dehydrogenase E1 component
BCAL2465	1.277	0.047	2.042	0.014	TetR family regulatory protein
BCAL2767	1.281	0.270	1.397	0.382	ArgF ornithine carbamoyltransferase
BCAL2782	1.373	0.237	1.166	0.668	PdxH pyridoxamine 5′-phosphate oxidase
BCAL3303	1.048	0.845	1.093	0.071	QueA *S*-adenosylmethionine:tRNA ribosyltransferase-isomerase
BCAM0820	2.621	0.004	2.253	0.002	Hybrid two-component system kinase-response regulator protein
BCAM0941	1.240	0.448	1.761	0.050	*gnd* 6-phosphogluconate dehydrogenase
BCAM1262	1.237	0.397	1.163	0.445	Dihydroxy-acid dehydratase
BCAM1415	1.183	0.665	1.315	0.177	AraC family regulatory protein
BCAM2738	1.213	0.147	1.649	0.022	IspH 4-hydroxy-3-methylbut-2-enyl diphosphate reductase
BCAS0223	1.251	0.202	1.993	0.030	AfcC fatty acid desaturase

**TABLE 2:** Expression changes of genes with a methylated GTWWAC motif in their promoter region in deletion mutants compared to wild type

Locus tag	J2315		K56-2		Gene function
	Fold change	*P* value	Fold change	*P* value	
BCAL0054	0.487	0.127	0.641	0.107	MerR family regulatory protein
BCAL0079	2.838	0.020	3.074	0.005	Rep ATP-dependent DNA helicase
BCAL0126	0.845	0.356	0.801	0.607	MotA chemotaxis protein
BCAL0162	0.139	0.634	0.133	0.478	GmhA phosphoheptose isomerase
BCAL0508	1.015	0.934	1.721	0.137	LpxL lipid A biosynthesis myristoyl acyltransferase
BCAL0709	1.599	0.104	0.763	0.430	LipB lipoate-protein ligase B
BCAL1556	1.611	0.171	1.690	0.006	RpiA ribose-5-phosphate isomerase A
BCAL2406	1.693	0.273	1.009	0.938	WabR putative glycosyltransferase
BCAL2415	2.819	0.006	6.029	0.001	PurT phosphoribosylglycinamide formyltransferase 2
BCAL2701	0.613	0.076	1.519	0.170	A*r*gD acetylornithine transaminase protein
BCAL2942	1.143	0.474	1.451	0.274	CysM cysteine synthase B
BCAM0076	1.630	0.112	1.358	0.051	TetR family regulatory protein
BCAM1362	1.959	0.025	1.516	0.004	Putative penicillin-binding protein
BCAS0258	1.247	0.451	1.141	0.438	GntR family regulatory protein

10.1128/mSphere.00455-20.5 FIG S5 Differential expression (volcano plots) of all genes with methylated promoter region in J2315 (left) and K56-2 (right) for which expression was quantified using qPCR. Cutoffs were drawn at fold changes −1.5 and 1.5 (blue) and at *P* value 0.05 (red). All genes outside these cutoffs were considered significantly up- or downregulated. Download FIG S5, TIF file, 0.3 MB. Copyright © 2020 Vandenbussche et al. 2020 Vandenbussche et al. This content is distributed under the terms of the Creative Commons Attribution 4.0 International license.

**FIG 7:**
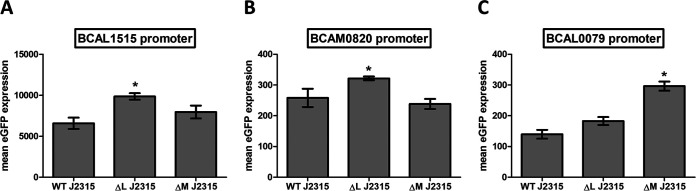
eGFP production in B. cenocepacia J2315 strains harboring a pJH2 plasmid that contains a BCAL1515 promoter-eGFP construct (A), a BCAM0820 promoter-eGFP construct (B), or a BCAL0079 promoter-eGFP construct (C). BCAL1515 and BCAM0820 are associated with methylation of the CACAG motif by DNA MTase BCAL3494, and BCAL0079 is associated with methylation of the GTWWAC motif by DNA MTase BCAL0992. *n* = 3; *, *P* < 0.05 compared to wild type; error bars represent the SEM. WT, wild type; ΔL, deletion mutant ΔBCAL3494; ΔM, deletion mutant ΔBCAM0992.

To confirm that the presence of methylation close to the −10 or −30/35 element influences transcription and therefore gene expression in B. cenocepacia, translational enhanced green fluorescent protein (eGFP) reporter fusions were constructed and eGFP production was quantified. The eGFP production in strains harboring different plasmids is shown in Fig. 7. As expected, the production of eGFP, driven by the promoters of genes BCAL1515, BCAM0820, and BCAL0079, was significantly (*P* = 0.001, *P* = 0.014, and *P* = 0.002, respectively) increased in the deletion mutant for which an upregulation of these genes was observed using qPCR experiments (Fig. 7).

### DNA methylation in the origin of replication.

**FIG 8:**
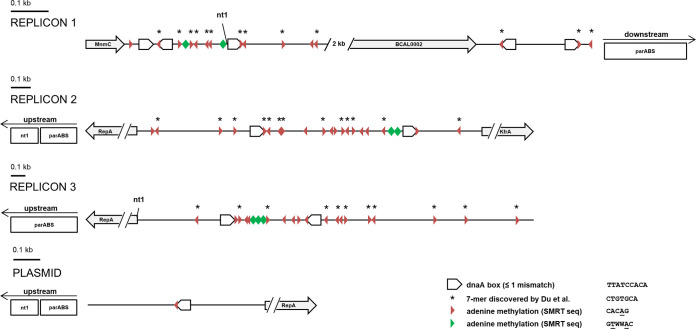
Methylation in the origin of replication of the different replicons in B. cenocepacia J2315. SMRT sequencing was used to detect methylated CACAG (red triangles) and GTWWAC (green triangles) motifs within these regions. DnaA boxes (TTATCCACA, consensus sequence of DnaA boxes in E. coli) are indicated in the figure. CACAG motifs were frequently found to be part of a previously discovered 7-mer (sense, CTGTGCA; antisense, TGCACAG) (34). The positions of these 7-mers are indicated with an asterisk. nt1, nucleotide 1; parABS genes, responsible for chromosome segregation in B. cenocepacia.

DNA methylation was detected in all origins of replication of B. cenocepacia (Fig. 8). Similar methylation patterns were observed in the origins of the different replicons. A previously discovered 7-mer (CTGTGCA) that can be found in all replication origins (34) contains a CACAG methylation motif on the antisense strand. This motif was also found at the 3′ end of almost every DnaA box. These boxes are bound by DnaA proteins, essential for DNA unwinding and chromosome replication initiation (35). Also, the GTWWAC motif was found in proximity to the replication origins; consequently, the origins in B. cenocepacia represent methylation-rich regions. Whereas methylated CACAG motifs were found throughout the origins of replication, the position of the GTWWAC methylation was unique in all replicons and at least two GTWWAC motifs were found in between two CACAG methylated DnaA boxes. In contrast to the origins of the three larger replicons, the origin of replication of the plasmid contained only one CACAG methylated DnaA box.

**FIG 9:**
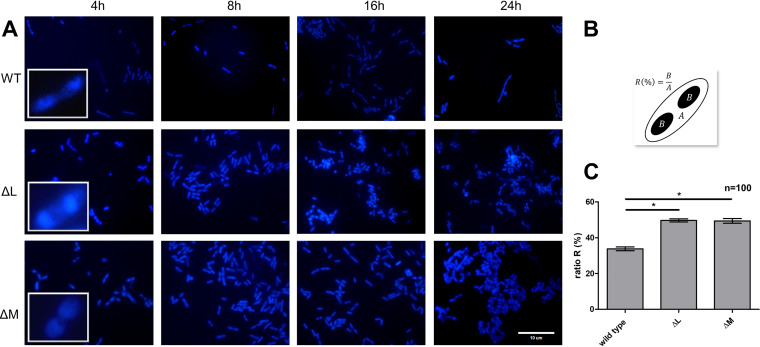
(A) DAPI staining of B. cenocepacia wild type and MTase mutants at 4 h, 8 h, 16 h, and 24 h. (B) The nucleoid size relative to the cell size (ratio, R) was calculated by dividing the surface area occupied by the nucleoid (section B) by the total surface area of the cell (section A). (C) Comparison of ratio R of wild type and deletion mutants (average over all time points). WT, wild type; ΔL, ΔBCAL3494; ΔM, ΔBCAM0992. *n* = 100 for each strain; error bars represent the SEM; *, *P* < 0.001 compared to wild type.

To assess the impact of DNA methylation within the replication origins on replication, we visualized the nucleoids in B. cenocepacia wild-type and mutant cells at different time points and calculated the relative nucleoid size (Fig. 9). In both ΔBCAL3494 (49.7%) and ΔBCAM0992 (48.7%), this ratio significantly differed from wild type (33.8%, *P* value of <0.001), which suggests that methylation of the replication origins influences DNA compaction. In general, this observation strengthens the proof of replication regulation by DNA methylation. We also investigated differences in cell morphology, but no differences in shape, structure, or size could be observed.

## DISCUSSION

Despite the growing knowledge of DNA methylation in prokaryotes (15), the role of DNA MTases in regulating gene expression in B. cenocepacia remains to be revealed. In the present study, we identified two DNA MTases (BCAL3494 and BCAM0992), and mutants in which these genes were deleted showed differences in biofilm formation and motility. When methylation was blocked by the DNA MTase inhibitor sinefungin (36), the same phenotypic differences were observed. SAM is important in cysteine metabolism (37), and while it cannot be ruled out that addition of sinefungin has an effect beyond DNA methylation, the observation that only differences in biofilm, cell aggregation, and motility were observed (i.e., the same phenotypes affected by MTase deletion) suggests this is not the case.

These findings demonstrate that epigenetic control of gene expression by MTases plays an important role in controlling certain phenotypes. Similar results have been reported in Salmonella enterica, where DNA methylation is crucial for optimal pellicle and biofilm production (38). The phenotypic changes in biofilm formation and cell clustering did not correlate with a significant decrease in virulence of B. cenocepacia in the G. mellonella infection model, suggesting DNA methylation is not essential for full virulence in B. cenocepacia.

Methylome analysis showed that mutants in which MTase ΔBCAL3494 or ΔBCAM0992 was inactivated lacked adenine methylation in specific motifs. MTase BCAL3494 was specifically linked to methylation of the CACAG motif, and MTase BCAM0992 was linked to methylation of the GTWWAC motif. This strategy of DNA methylation analysis, in which the methylome of strains lacking MTases is determined, has been used in various bacteria, as it is an effective way to find associations between predicted MTases and genome-wide methylation motifs (39, 40). For example, several methylation motifs were identified in Burkholderia pseudomallei, including motifs CACAG and GTWWAC (41). Two of the B. pseudomallei MTases (M.BpsI and M.BpsII) are homologous to the B. cenocepacia MTases BCAL3494 and BCAM0992. In Ralstonia solanacearum, an important plant pathogen that is phylogenetically related to B. cenocepacia, the GTWWAC methylation motif cooccurs with the respective homolog of the BCAM0992 MTase, whereas a BCAL3494 MTase homolog and methylation of CACAG are absent (42). As in B. cenocepacia, the BCAM0992 homolog in R. solanacearum is an orphan DNA MTase. Analysis of cytosine methylation suggests that cytosine is more likely methylated at random instead of at specific motifs and is likely not having a major regulatory function. Also, GC-rich genomes complicate the search for specific cytosine motifs.

Previous epigenetic research demonstrated that in most cases, there is a negative correlation between methylation in promoters and transcription (43). To uncover the role of DNA methylation in regulation of B. cenocepacia gene expression, all methylated motifs in promoter regions were identified. The data obtained in the present study indicate that gene expression was upregulated in DNA MTase mutants, suggesting that adenine DNA methylation in B. cenocepacia affects gene expression by a mechanism inhibiting transcription. In both prokaryotes and eukaryotes, adenine and cytosine methylation are involved in blocking (or enhancing) the binding of RNA polymerase to DNA (15, 21, 44), and especially methylation near the −10 and −30/35 elements in the promoter region may affect RNA polymerase binding (45). We found that also in B. cenocepacia, methylated motifs (CACAG and GTWWAC) are found close to or in these elements.

BCAM0820, upregulated in the J2315 and K56-2 ΔBCAL3494 mutant, is a two-component response regulator, the first gene of an operon homologous to the Wsp chemosensory system involved in biofilm formation in Pseudomonas aeruginosa (46). BCAM0820 is homologous to WspR but lacks the diguanylate cyclase domain. During an experimental evolution study in which B. cenocepacia HI2424 biofilms were grown on beads, mutations within the *wsp* gene cluster occurred in different clones; these were associated with increased pellicle formation and increased biofilm formation on beads. This demonstrates that the Wsp cluster is involved in pellicle formation in B. cenocepacia (47, 48), and the upregulation of BCAM0820 could explain the differences in pellicle and biofilm formation between the wild-type strains and the ΔBCAL3494 deletion mutants observed in the present study. Interestingly, BCAL1515, encoding 2-oxoglutarate dehydrogenase (SucA) and upregulated in ΔBCAL3494, also acquired mutations in the course of the experimental evolution study (48), but the role of this gene in biofilm formation has not been further explored. BCAL0079, upregulated in the ΔBCAM0992 mutants, is annotated as a DNA helicase gene (*rep*). Besides unwinding DNA during DNA replication, Rep plays a role in swimming motility in E. coli (49). The reduced motility observed in the ΔBCAM0992 mutants suggests that Rep may also affect motility in B. cenocepacia, although this remains to be confirmed.

**FIG 10:**
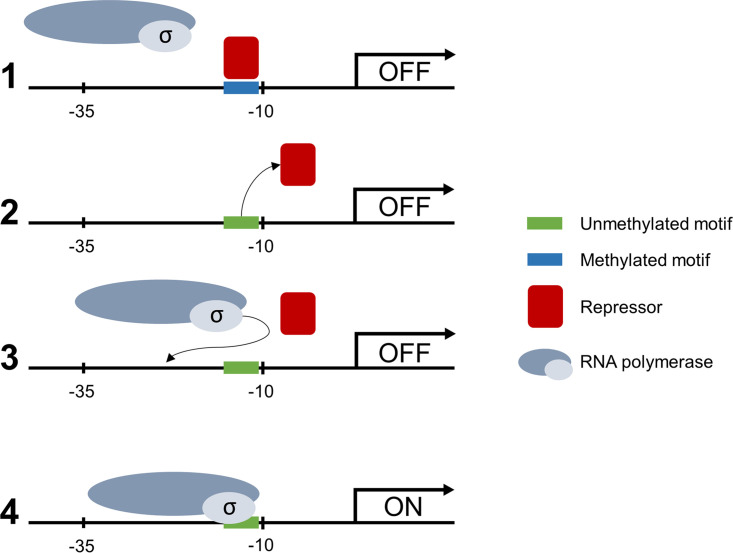
Proposed mechanism of regulation of gene expression in B. cenocepacia. (1) Methylated motifs in the promoter region of the gene are bound by a TF, acting as repressor (OFF state). (2) In the absence of methylation in the promoter region, the TF dissociates from the motif and vacates the promoter region. (3) The sigma factor is no longer sterically hindered by a repressor and is able to bind to the promoter region. (4) RNA polymerase can access the promoter region and start transcription of the gene (ON state).

Measurement of eGFP production in translational fusion mutants revealed that mutants with constructs containing the BCAL1515, BCAM0820, or BCAL0079 promoter showed a significant increase in eGFP production compared to wild type, thereby supporting our hypothesis of gene expression regulation by DNA methylation. *In silico* analyses predict that sequences containing methylation motifs are similar to binding sites of TF in E. coli K-12, and it is plausible that these sequences are also part of TF binding sites in B. cenocepacia, allowing us to propose a possible mechanism of gene expression regulation (Fig. 10). TFs that bind close to the −10 and −35 region often act as transcriptional repressors (50). Therefore, a methylated promoter region could promote binding of a repressor (51) and sterically hinder RNA polymerase (OFF state), whereas an absence of methylation could lead to binding of RNA polymerase and initiation of transcription (ON state).

The role of DNA methylation in prokaryotes in multifaceted. Besides gene expression regulation and a role in DNA mismatch repair in Gram-positive bacteria (52), DNA methylation has also been implicated in the coordination of replication initiation. Results of the present study seem to confirm this, as the *rep* gene, necessary for replication, is under DNA-methylation-mediated epigenetic control. In E. coli, GATC motifs, omnipresent in the replication origin, are prone to adenine methylation. The motifs are found within DnaA boxes, essential for binding of the DnaA protein and initiation of replication. The methylation state of each of these GATC motifs changes the affinity of DnaA and sequestering-protein SeqA for the DnaA box. Immediately after replication, GATC motifs are hemimethylated, which leads to sequestration of the DnaA boxes by SeqA and prevents the reinitiation of DNA replication (21). The occurrence of methylated motifs in the vicinity of the origins of replication of the four replicons in B. cenocepacia was studied to check for a link between DNA methylation and coordination of the replication process. An enrichment of the CACAG motif was observed in the origin of replication of all replicons. The motif was part of a bigger sequence that has previously been reported as a recurring 7-mer (34), without known function. In addition, the origin of replication of the different replicons showed high similarities in methylation patterns, and both mutants appeared to have a larger nucleoid size with respect to the total cell size compared to wild type, raising the possibility of replication coordination by DNA methylation. The importance of adenine methylation in DNA replication has been studied in other bacteria besides E. coli, and for example in Vibrio cholerae adenine methylation is also frequently found in the origin of replication of both chromosomes (53), just like in B. cenocepacia. Although the lack of differences in growth between B. cenocepacia wild type and MTase mutants during exponential phase suggests the impact of DNA methylation on replication is limited, the K56-2 ΔBCAL3494 mutant did reach maximal optical density in stationary phase a bit earlier than the wild type (see Fig. S2 in the supplemental material). This is in line with the observation that the promoter of *dnaA* (BCAL0423) was methylated at multiple positions and under transcriptional control of the BCAL3494 MTase (Fig. 6). Combined, these data suggest that DNA methylation contributes to coordinating the replication process in B. cenocepacia K56-2. In the slower-growing J2315 strain, such difference in maximal optical density was not observed, and in this strain, expression of *dnaA* was found not to be transcriptionally controlled by BCAL3494, suggesting that coordination of replication is strain dependent.

In conclusion, we have demonstrated that DNA methylation plays a role in regulation of gene expression in B. cenocepacia. DNA MTases BCAL3494 and BCAM0992 are essential for methylation of the B. cenocepacia genome and are responsible for methylation of base motifs CACAG and GTWWAC, respectively. The absence of methylation resulted in an upregulation of certain genes with methylatable promoter regions, including BCAM0820 and BCAL0079, genes of which their function can be linked to the observed biofilm- and motility-affected phenotypes. Finally, recurrent methylation patterns were detected in all origins of replication, which suggests an additional role of DNA methylation in replication regulation.

## MATERIALS AND METHODS

### Strains and culture conditions.

All strains and plasmids used in this study are listed in Table 3. B. cenocepacia strains were cultivated in phosphate-buffered mineral medium (2.00 g/liter NH_4_Cl, 4.25 g/liter K_2_HPO_4_·3H_2_O [ChemLab], 1.00 g/liter NaH_2_PO_4_·H_2_O, 0.10 g/liter nitriloacetic acid, 0.0030 g/liter MnSO_4_·H_2_O, 0.0030 g/liter ZnSO_4_·7H_2_O, 0.0010 g/liter CoSO_4_·7H_2_O, 0.20 g/liter MgSO_4_·7H_2_O, 0.012 g/liter FeSO_4_·7H_2_O [Sigma-Aldrich], 5 g/liter yeast extract [Lab M], 2 g/liter Casamino Acids [BD Biosciences], and 5 g/liter glycerol [Scharlab]). LB medium (Luria-Bertani medium with 5 g/liter NaCl; Sigma-Aldrich) was used for maintenance of E. coli strains and during specific stages of the gene deletion procedure (see below) where antibiotic selection with tetracycline (250 μg/ml) (Sigma-Aldrich) was desired. Prior to phenotypic experiments, liquid overnight cultures were grown in a shaker incubator (100 rpm) at 37°C.

**TABLE 3:** Bacterial strains and plasmids

Strain or plasmid	Description	Abbreviation	Source or reference(s)
B. cenocepacia			
J2315	CF sputum isolate	WT J2315	LMG16656
J2315 ΔBCAL3494	BCAL3494 MTase deletion mutant	ΔL J2315	This study
J2315 ΔBCAM0992	BCAM0992 MTase deletion mutant	ΔM J2315	This study
J2315 ΔBCAL3494 pJH2	BCAL3494 MTase mutant with empty pJH2 vector	ΔL pJH2 J2315	This study
J2315 ΔBCAL3494 pJH2 + BCAL3494	BCAL3494 MTase complemented deletion mutant	*c*ΔL pJH2 J2315	This study
J2315 ΔBCAM0992 pJH2	BCAM0992 MTase mutant with empty pJH2 vector	ΔM pJH2 J2315	This study
J2315 ΔBCAM0992 pJH2 + BCAM0992	BCAM0992 MTase complemented deletion mutant	*c*ΔM pJH2 J2315	This study
K56-2	CF sputum isolate	WT K56-2	LMG 18863
K56-2 ΔBCAL3494	BCAL3494 MTase deletion mutant	ΔL K56-2	This study
K56-2 ΔBCAM0992	BCAM0992 MTase deletion mutant	ΔM K56-2	This study
K56-2 ΔBCAL3494 pJH2	BCAL3494 MTase mutant with empty pJH2 vector	ΔL pJH2 K56-2	This study
K56-2 ΔBCAL3494 pJH2 + BCAL3494	BCAL3494 MTase complemented deletion mutant	*c*ΔL pJH2 K56-2	This study
K56-2 ΔBCAM0992 pJH2	BCAM0992 MTase mutant with empty pJH2 vector	ΔM pJH2 K56-2	This study
K56-2 ΔBCAM0992 pJH2 + BCAM0992	BCAM0992 MTase complemented deletion mutant	*c*ΔM pJH2 K56-2	This study

E. coli			
DH5α	Maintenance of replicative plasmids		Lab stock
One Shot PIR2	Maintenance of suicide plasmids with ori_R6K_	PIR2	ThermoFisher

Plasmids			
pGPI-SceI-XCm	Suicide plasmid, Tp^r^, Cm^r^, I-SceI restriction site, ori_R6K_	pGPI	57, 58
pDAI-SceI-SacB	Replicative plasmid, Tet^r^, I-SceI nuclease, counterselectable marker SacB, ori_pBBR1_	pDAI	57, 58
pRK2013	Helper plasmid, Km^r^, ori_colEI_	pRK	57, 58
pJH2	Broad-range translational fusion vector, Cm^r^, fluorescent marker eGFP: complementation of ΔBCAL3494		59
pSCrhaB2	Broad-range translational fusion vector, Tp^r^, rhaR, rhaS-P_rhaB_, ori_pBBr1_: complementation of ΔBCAM0992		60
pGPI + BCAL3494 upstream sequence	pGPI-SceI-XCm with ligated upstream sequence BCAL3494, used during deletion	pGPI_UL_	This study
pGPI + BCAL3494 upstream and downstream sequence	pGPI-SceI-XCm with ligated upstream and downstream sequence BCAL3494, used during deletion	pGPI_UL-DL_	This study
pGPI + BCAM0992 upstream sequence	pGPI-SceI-XCm with ligated upstream sequence BCAM0992, used during deletion	pGPI_UM_	This study
pGPI + BCAM0992 upstream and downstream sequence	pGPI-SceI-XCm with ligated upstream and downstream sequence BCAM0992, used during deletion	pGPI_UM-DM_	This study
pJH2 + BCAL3494 sequence	Fusion vector with ligated BCAL3494 sequence, used for complementation	pJH2_L3494_	This study
pSCrhaB2 + BCAM0992 sequence	Fusion vector with ligated BCAM0992 sequence, used for complementation	pSCrhaB2_M0992_	This study

### Selection of DNA MTase genes—*in silico*.

The REBASE Genome database was used to allocate all known DNA MTase genes in the B. cenocepacia J2315 and K56-2 genomes (54). The Artemis Genome Browser and Annotation Tool (Sanger) allowed visualization of the genomic context of these genes (55). NCBI BLAST was used to screen for conservation of the genes within the *Burkholderia* genus using default search parameters (56) (search mode, BLASTn; E cutoff value, <1E−5).

### Construction of deletion mutants.

All primers used for construction and complementation of the deletion mutants are listed in Table S4 in the supplemental material. The procedure is an adapted allelic replacement approach, using a suicide plasmid with an SceI endonuclease recognition site (57, 58). The suicide plasmid, containing DNA fragments of regions flanking the target gene, is integrated into the B. cenocepacia genome by homologous recombination. Introducing a second plasmid that carries SceI endonuclease genes into B. cenocepacia results in a lethal genomic strand break. Another homologous recombination event allows the bacteria to repair the break with a 50% chance of resulting in a gene deletion. Deletion mutants ΔBCAL3494 and ΔBCAM0992 were constructed in both B. cenocepacia J2315 and K56-2.

10.1128/mSphere.00455-20.10 TABLE S4 Overview of all primers used for construction and complementation of gene deletion mutants, qPCR experiments, and construction of translational eGFP reporter fusion plasmids. Download Table S4, DOCX file, 0.02 MB. Copyright © 2020 Vandenbussche et al. 2020 Vandenbussche et al. This content is distributed under the terms of the Creative Commons Attribution 4.0 International license.

BCAL3494 was deleted together with neighboring gene BCAL3493, as well as BCAL3488 to BCAL3492 (encoding hypothetical proteins). Targeting BCAL3494 alone was not feasible because regions flanking BCAL3494 contain multiple recognition sites for endonucleases used during construction of the deletion mutants, and digestion of these regions would be inevitable (Fig. S1).

E. coli One Shot PIR2 cells (Thermo Fisher), expressing λ *pir*, were used for transformation, replication, and maintenance of the suicide plasmid during construction of deletion mutants. Thawed cells were immediately exposed to a heat shock transformation procedure, after which they were transferred to SOC (Super Optimal broth with Catabolite repression) medium for recovery. For plasmid selection, the phosphate-buffered mineral medium was supplemented with one or more of the following antibiotics: trimethoprim (Ludeco; 50 μg/ml for initial screening in E. coli, 200 μg/ml when plasmid is introduced in B. cenocepacia), chloramphenicol (400 μg/ml), gentamicin (50 μg/ml), kanamycin (50 μg/ml), and ampicillin (200 μg/ml) (Sigma-Aldrich).

### Construction of plasmids for complementation.

To ensure that phenotypes were solely caused by the deletion of DNA MTases, deletion mutants were complemented. The primers used for construction of plasmids used for complementation are listed in Table S4. Plasmids pJH2 and pSCrhaB2 were used for complementation of ΔBCAL3494 (*c*ΔBCAL3494) and ΔBCAM0992 (*c*ΔBCAM0992), respectively. The genomic sequences of the DNA MTase genes were PCR amplified and subsequently cloned into the plasmids. BCAL3494 was amplified, including its own regulatory region (approximately 250 nucleotides upstream of the transcription start site), into pJH2, which does not have a promoter associated with its multiple cloning site (59). BCAM0992 does not have its own upstream promoter; therefore, it was cloned into pSCrhaB2, which contains a rhamnose-inducible promoter (60). Complemented mutant strains were subjected to the same phenotypic tests as the deletion mutants and wild-type B. cenocepacia. For strain *c*ΔBCAM0992, the phosphate-buffered mineral medium was supplemented with 0.2% rhamnose.

### Biofilm and clustering experiments.

Biofilms were grown in plastic U-shaped 96-well microtiter plates in phosphate-buffered medium at 37°C, starting from 200-μl/well planktonic overnight cultures with an optical density (OD) of 0.05 (590 nm). After 4 h static incubation, all wells were rinsed with physiological saline (PS; 0.9% NaCl in water), thereby removing all unattached planktonic cells. Wells were refilled with 200 μl medium and incubated for an additional 20 h. Where appropriate, biofilms were stained with LIVE/DEAD (SYTO9/propidium iodide; Invitrogen) to visualize the bacteria and distinguish live and dead cells (61). Pellicle formation was determined in glass tubes. Cultures were grown statically for 24 h, after which adhering pellicles were stained and quantified with crystal violet (62). Cell clustering, already shown to be correlated with pellicle formation, was determined with flow cytometry (Attune NxT flow cytometer; Thermo Fisher) (63). Forward scatter (FSC), a value for particle size, and side scatter (SSC), a value for particle complexity, were measured for each particle present in the bacterial suspension and visualized in scatterplots. After analysis of these graphs, the main cell population was gated (gate ranging from approximately 10^3^ to 10^5^ for both FSC and SSC), and detected events larger and more complex than the gate were considered clustered (Fig. S6).

10.1128/mSphere.00455-20.6 FIG S6 Quantification of the number of clusters in wild-type J2315 (left) and ΔBCAL3494 J2315 (right). SSC, side scatter; FSC, forward scatter; green circle indicates clusters; red circle indicates main population (ranging from approximately 10^3^ to 10^5^ for both FSC and SSC). Download FIG S6, TIF file, 0.8 MB. Copyright © 2020 Vandenbussche et al. 2020 Vandenbussche et al. This content is distributed under the terms of the Creative Commons Attribution 4.0 International license.

### Motility experiments.

Petri dishes containing phosphate-buffered mineral medium with agar concentrations of 0.3% and 0.5% were used for assessment of swimming and swarming motility, respectively. One microliter of cultures with an OD of 0.1 was spotted on the agar plates. Diameters were measured after 24 h (strain K56-2) or 32 h (strain J2315).

### DNA MTase inhibition with sinefungin.

A stock solution of the DNA MTase inhibitor sinefungin (Sigma-Aldrich) was prepared (10 mg/ml) (31), aliquoted, and immediately frozen at −20°C to prevent degradation. Cells were grown for 24 h in sinefungin-supplemented medium (50 μg/ml) and used as inoculum for an overnight culture, also in sinefungin-supplemented medium. This allowed the DNA MTase inhibitor to have an effect during several growth cycles. Then, biofilm formation and motility of sinefungin-treated cells were assessed as described above in medium supplemented with 50 μg/ml sinefungin.

### Virulence assay.

Galleria mellonella larvae (TruLarv; BioSystems Technology) were injected in the left proleg with 10 μl bacterial suspension (set to an OD of 0.2 and diluted 100-fold before injection). A physiological saline solution was used as control. After infection, the larvae were incubated at 37°C, upon which viability was checked every 24 h. Eight larvae were included in each group.

### Genomic DNA extraction.

Prior to DNA extraction, planktonic strains were grown overnight in a shaker incubator (100 rpm) at 37°C. Biofilm cells were grown as described above. Next, cells were harvested and genomic DNA (gDNA) was extracted using the Wizard Genomic DNA purification kit (Promega). Quantification was performed with a BioDrop μLITE (BioDrop) spectrophotometer.

### SMRT sequencing.

To determine the methylome of B. cenocepacia, gDNA extracts were analyzed with single-molecule real-time (SMRT) sequencing technology. gDNA samples of both wild-type and mutant strains were run on a Pacific Biosciences Sequel System (250× coverage) according to the manufacturer’s guidelines. Library preparations were multiplexed as data output of approximately 2 Gb per genome was expected, and a single SMRT Sequel cell provides up to 6 Gb data. Initial data output was processed with SMRT Link software (Pacific Biosciences). Identification of the modified bases and analysis of the methylated motifs were performed with the Base Modification and Motif Analysis application (SMRT Link v6.0; Pacific Biosciences). In-depth data analysis was performed with CLC Workbench Genomics (v11.0.1; Qiagen). Differential analysis between wild type and mutants was performed to identify methylation motifs specifically associated with certain DNA MTases. Previously predicted promoter regions and transcription start sites of B. cenocepacia were used to determine the methylation profile of regulatory regions (64). Virtual Footprint software (promoter analysis mode, default search parameters) was used to assess similarity of the methylation motifs to known TF binding sites (65).

### qPCR.

To evaluate the impact of DNA methylation in promoter regions on gene expression, qPCR was performed on all genes that had a methylated promoter region in wild-type B. cenocepacia but an absence of methylation in the promoter region in one of the deletion mutants. All hypothetical genes and genes with unknown function, as well as genes with low innate expression level, were excluded from testing. The primers used for qPCR are listed in Table S4. First, all strains were grown to an OD of 0.6 in phosphate-buffered medium, after which they were pelleted by centrifugation and frozen at −80°C. Next, RNA was extracted using the RiboPure bacterial extraction kit (Invitrogen), followed by a DNase treatment to remove trace quantities of gDNA. Quantification and measurement of RNA purity of the extracts were performed with a BioDrop μLITE (BioDrop). Subsequently, cDNA was synthesized, using 500 ng RNA per reaction, with a reverse transcriptase kit (high-capacity cDNA RT kit; Applied Biosystems). Per qPCR mixture, 2 μl template cDNA was mixed with 10 μl GoTaq qPCR master mix, 0.6 μl qPCR primer mix (10 μg/ml), and 7.4 μl nuclease-free water according to the GoTaq qPCR master mix (Promega) protocol. Samples were run on a CFX96 Real-Time System C1000 Thermal Cycler (Bio-Rad), and output data were processed with Bio-Rad CFX Manager 3.1 software. The baseline threshold was set to a defined 100 relative fluorescence units. Obtained quantification cycle (*C_q_*) values were normalized to reference gene *rpoD* (BCAM0918) of which the expression was stable across all samples; differences from wild type were calculated (ΔΔ*C_q_*) and log-transformed. Volcano plots were used to plot the negative logarithm of statistical *P* values against log_2_ fold changes (Fig. S5).

### Construction of translational eGFP reporter fusions and measurement of eGFP production.

Genes with methylated promoter regions that showed a significant upregulation of gene expression in one of both mutant strains were selected for eGFP experiments. Translational eGFP reporter fusion plasmids were constructed by cloning the regulatory regions of the genes, comprising 60 to 390 nucleotides upstream of the transcription start site, into vector pJH2. The insert is cloned right in front of the eGFP gene and contains an ATG start codon at the 3′ end, in frame with the codon sequence of the gene. All primers used for amplification of the regulatory regions and screening of pJH2 with correct insert length are listed in Table S4. The plasmids were transferred to B. cenocepacia J2315 and K56-2 by triparental mating. Exconjugants were grown on selective plates (LB medium supplemented with 200 μg/ml chloramphenicol and 50 μg/ml gentamicin) and PCR screened to confirm the presence of the insert. Constructs carrying genes BCAL2415, BCAL2465, and BCAM1362 repeatedly failed to be transferred to B. cenocepacia and were not included in further experiments. Fluorescent signals of eGFP production in wild-type and mutant strains were measured by flow cytometry (Attune NxT flow cytometer; Thermo Fisher) (59).

### Nucleoid staining and surface quantification.

To stain the nucleoids of B. cenocepacia, 4′,6-diamidino-2-phenylindole (DAPI) (Thermo Fisher) was used. In brief, cells were grown for various times, heat fixed on glass slides, stained with a DAPI solution (3 μg/ml) for 5 min, and visualized with fluorescence microscopy (EVOS FL Auto Imaging System; Thermo Fisher). To calculate nucleoid and total cell surfaces, we used the thresholding function of the Java-based processing application ImageJ (LOCI, University of Wisconsin).

### Data analysis and statistics.

Statistical analysis was performed using SPSS Statistics v. 25 software. All tests and experiments were run in triplicate unless otherwise mentioned. Normality of data was verified with a Shapiro-Wilk test. To check for significant differences between data, normally distributed data were subjected to a *t* test or one-way analysis of variance (ANOVA) test, not normally distributed data to a nonparametric Mann-Whitney U-test. A log rank (Mantel-Cox) test was used to assess G. mellonella data. Resulting *P* values smaller than 0.05 were reported as statistically significant.
