# Supplementary figures and images for: DNA Methylation Epigenetically Regulates Gene Expression in Burkholderia cenocepacia and Controls Biofilm Formation, Cell Aggregation, and Motility
Source: mSphere. 2020 Jul 15;5(4):e00455-20. doi: 10.1128/mSphere.00455-20 (PMC7364216; doi:10.1128/mSphere.00455-20)

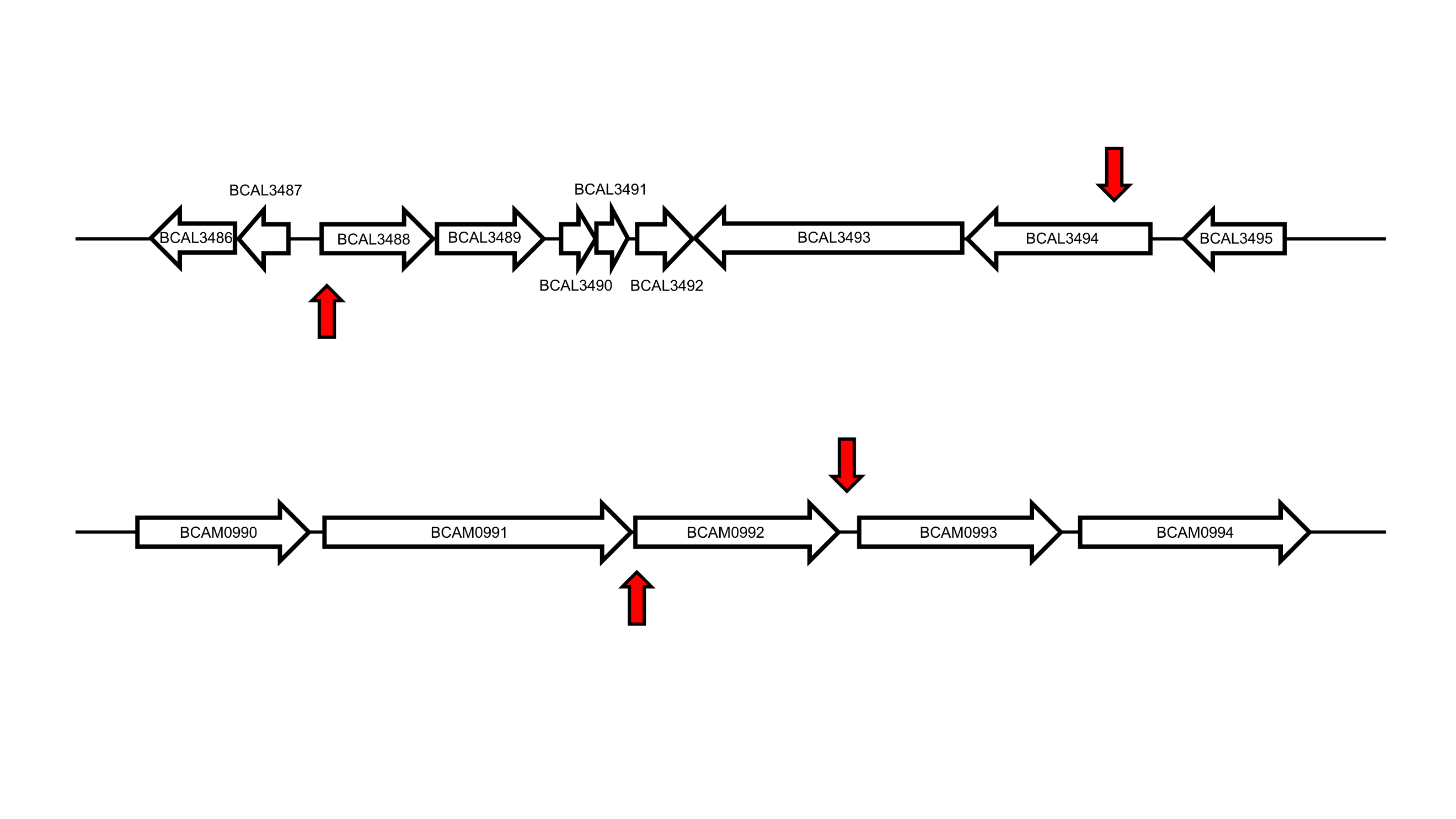

Supplement: FIG S1 [file mSphere.00455-20-sf001.tif]

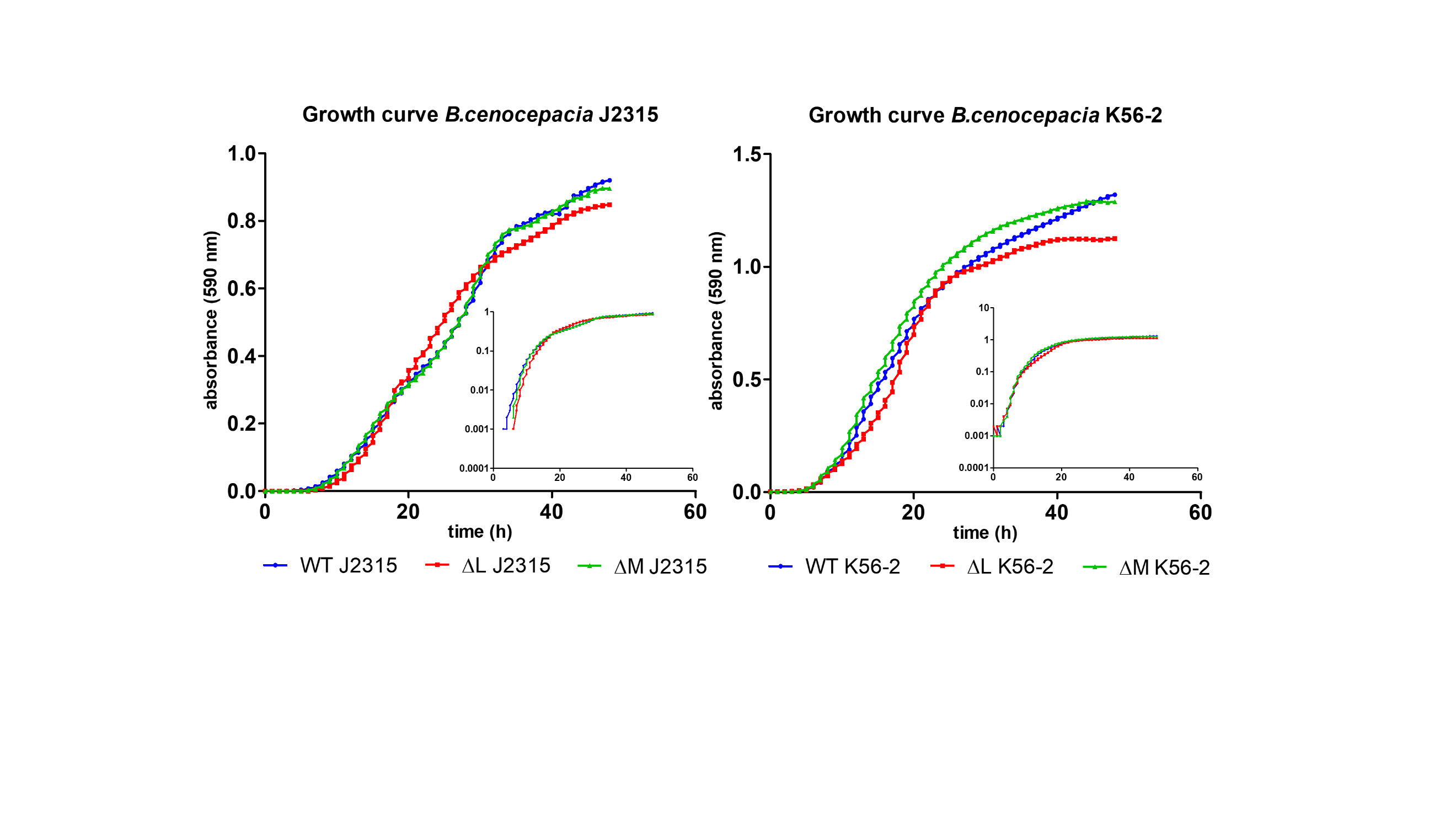

Supplement: FIG S2 [file mSphere.00455-20-sf002.tif]

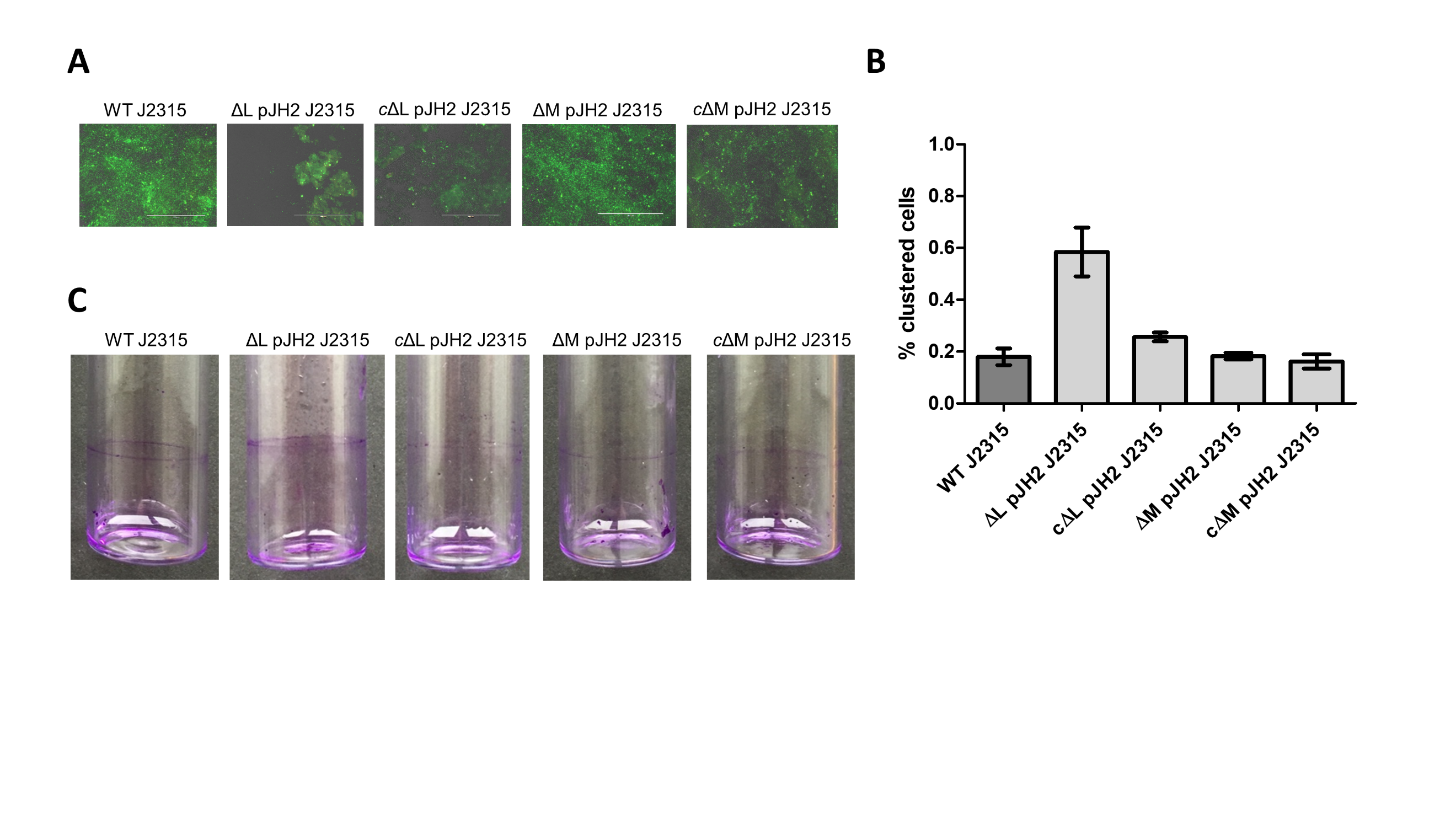

Supplement: FIG S3 [file mSphere.00455-20-sf003.tif]

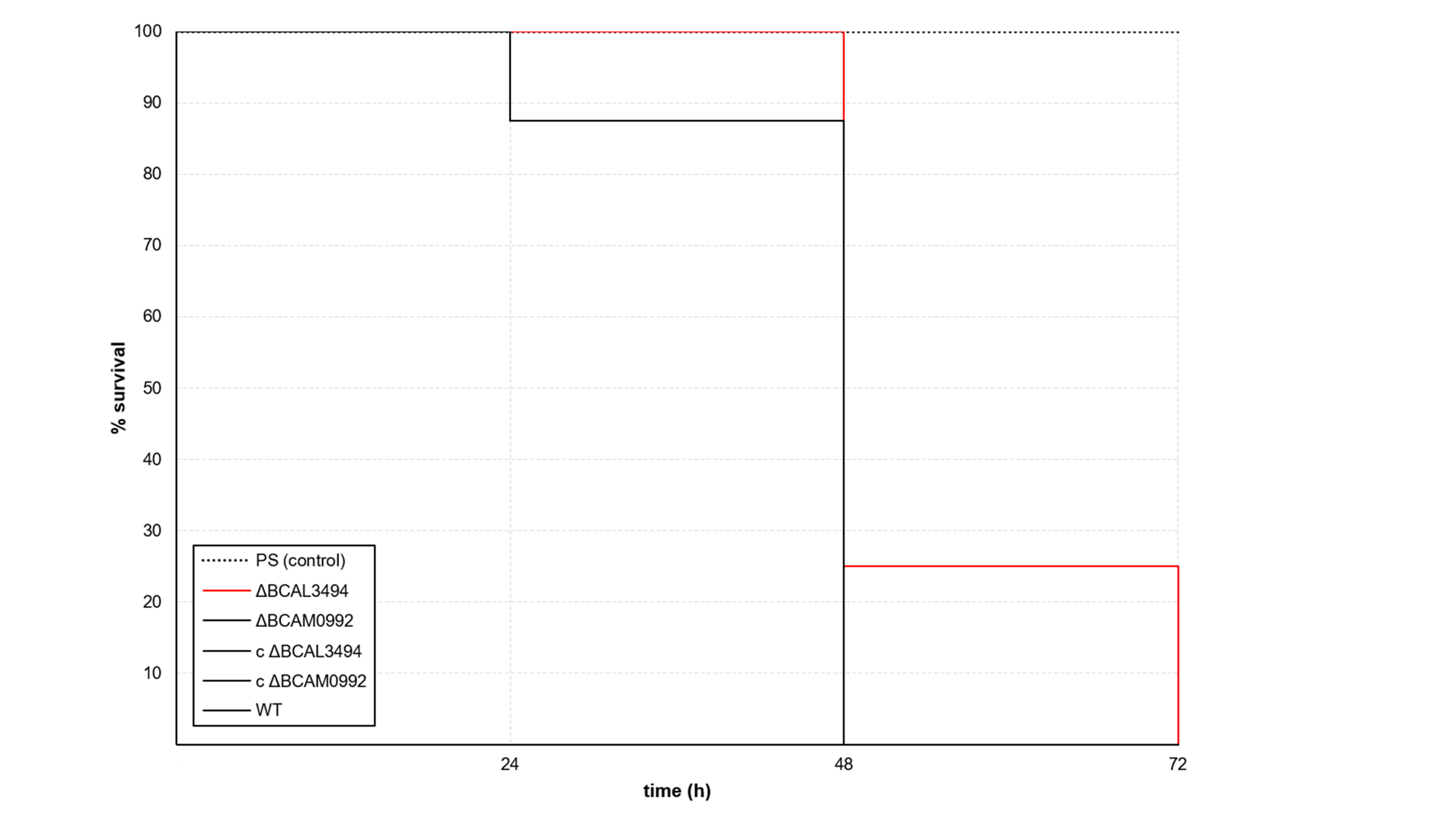

Supplement: FIG S4 [file mSphere.00455-20-sf004.tif]

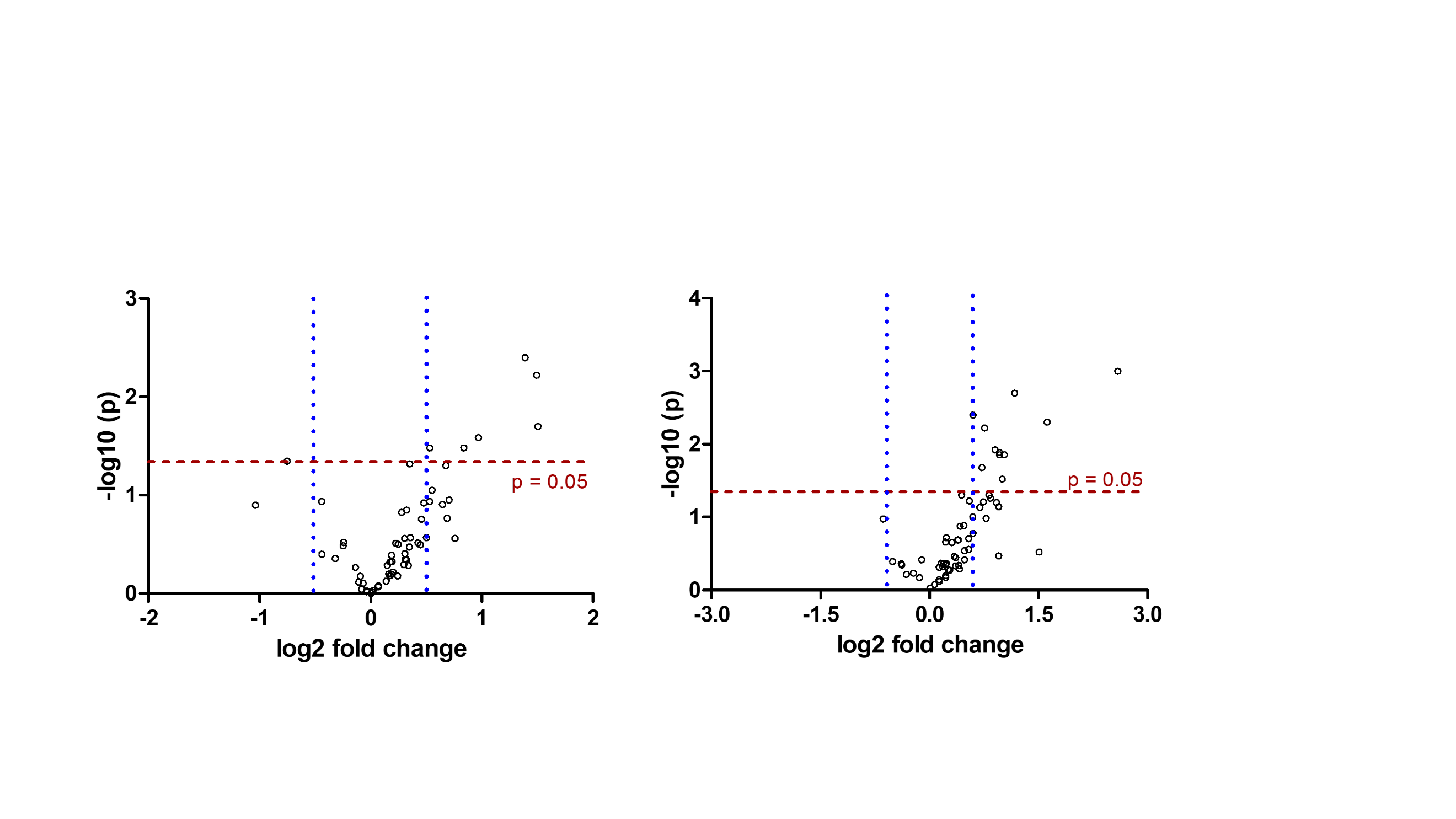

Supplement: FIG S5 [file mSphere.00455-20-sf005.tif]

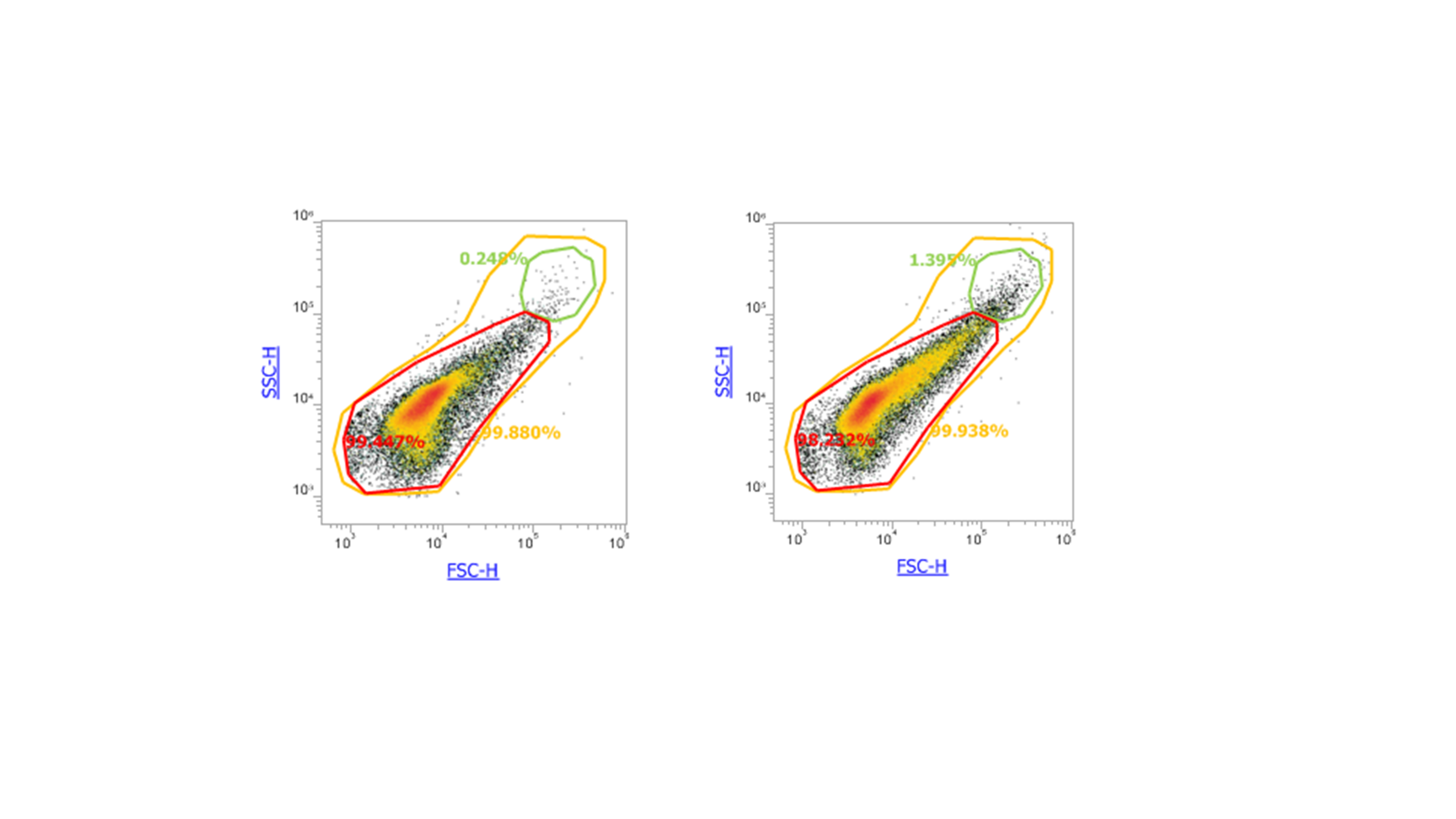

Supplement: FIG S6 [file mSphere.00455-20-sf006.tif]
