# Supplementary material for: DNA Methylation Epigenetically Regulates Gene Expression in Burkholderia cenocepacia and Controls Biofilm Formation, Cell Aggregation, and Motility
Source: mSphere. 2020 Jul 15;5(4):e00455-20. doi: 10.1128/mSphere.00455-20 (PMC7364216; doi:10.1128/mSphere.00455-20)
Supplement: TABLE S1 [file mSphere.00455-20-st001.docx]

| Locus Tag | Name | Location | Predicted type MTase (1) | Widely distributed in genus *Burkholderia* * | | Gene expression (2) |
| --- | --- | --- | --- | --- | --- | --- |
| BCAL3494 | M.BceJI | CHR 1 | Type III | | ✓ | Exponential phase |
| BCAM1036 | M.BceJII | CHR 2 |  | |  | Permantly low |
| pBCA072 | M.BceJIII | PLASMID |  | |  | Permantly low |
| BCAM0992 | M.BceJIV | CHR 2 |  | | ✓ | Exponential phase |
| BCAL0178 | M.BceJ178P | CHR 1 | Type II | |  | Exponential phase |
| BCAL0414 | M.BceJ414P | CHR 1 | Type I | |  | Exponential phase |

| Motif | Methylation | Strand | Called modified motifs * (%) | Methylation of motif by |
| --- | --- | --- | --- | --- |
| CACAG | 6mA | F | 6834/6836 (99.9) | **BCAL3494** |
| GTWWAC | 6mA | F+R | 961/982 (97.8) | **BCAM0992** |
| GCGGCCGC | 4mC | F+R | 1738/6850 (25.3) | unknown |

References

1. Roberts RJ, Vincze T, Posfai J, Macelis D. REBASE –– a database for DNA restriction and modification: enzymes, genes and genomes. Nucleic Acids Res. 2015;43:D298-299.

2. Kiekens S, Sass A, Van Nieuwerburgh F, Deforce D, Coenye T. The Small RNA ncS35 Regulates Growth in Burkholderia cenocepacia J2315. mSphere. 2018;3(1):1–16.
