# Supplementary material for: DNA Methylation Epigenetically Regulates Gene Expression in Burkholderia cenocepacia and Controls Biofilm Formation, Cell Aggregation, and Motility
Source: mSphere. 2020 Jul 15;5(4):e00455-20. doi: 10.1128/mSphere.00455-20 (PMC7364216; doi:10.1128/mSphere.00455-20)
Supplement: TABLE S2 [file mSphere.00455-20-st002.docx]

|  | Locus tag | Gene function | | | Methylated in K56-2 | |
| --- | --- | --- | --- | --- | --- | --- |
|  |  | **CACAG motif** | | |  | |
|  | **Intermediary metabolism** | | | | | |
| 1 | BCAL0627 | putative hydrolase | | | + | |
| 2 | BCAL1059 | ArgD bifunctional N-succinyldiaminopimelate-aminotransferase/acetylornithine transaminase protein | | | + | |
| 3 | BCAL1086 | putative lipoprotein | | | + | |
| 4 | BCAL1249 | putative PHB depolymerase | | | + | |
| 5 | BCAL1515 | SucA 2-oxoglutarate dehydrogenase E1 component | | | + | |
| 6 | BCAL1762 | acetyltransferase (GNAT) family protein | | | + | |
| 7 | BCAL1796 | putative saccharopine dehydrogenase | | | + | |
| 8 | BCAL1924 | MoeA3 molybdopterin biosynthesis protein | | | + | |
| 9 | BCAL2767 | ArgF ornithine carbamoyltransferase | | | + | |
| 10 | BCAL2782 | PdxH pyridoxamine 5'-phosphate oxidase | | | + | |
| 11 | BCAL2796 | benzoylformate decarboxylase | | | + | |
| 12 | BCAM0941 | *gnd* 6-phosphogluconate dehydrogenase | | | + | |
| 13 | BCAM1128 | putative glycosyl transferase family protein | | | + | |
| 14 | BCAM1204 | DadX alanine racemase | | | + | |
| 15 | BCAM1262 | IlvD dihydroxy-acid dehydratase | | | + | |
| 16 | BCAM1353 | *ald* alanine dehydrogenase | | | + | |
| 17 | BCAM1459 | AtoE short-chain fatty acid transporter | | | + | |
| 18 | BCAM1485 | ornithine cyclodeaminase | | | + | |
| 19 | BCAM2380 | putative D-isomer specific 2-hydroxyacid dehydrogenase | | | + | |
| 20 | BCAM2738 | IspH 4-hydroxy-3-methylbut-2-enyl diphosphate reductase | | | + | |
| 21 | BCAS0223 | AfcC putative fatty acid desaturase | | | + | |
| 22 | BCAS0257 | putative acetyltransferase | | | + | |
| 23 | BCAS0324 | sugar ABC transporter ATP-binding protein | | | + | |
|  | **Regulation** | | | | | |
| 1 | BCAL0003 | MarR family regulatory protein | | | + | |
| 2 | BCAL0003 | MarR family regulatory protein | | | + | |
| 3 | BCAL1457 | LysR family regulatory protein | | | + | |
| 4 | BCAL1477 | LysR family regulatory protein | | | + | |
| 5 | BCAL2443 | GntR family regulatory protein | | | + | |
| 6 | BCAL2465 | TetR family regulatory protein | | | + | |
| 7 | BCAL2465 | TetR family regulatory protein | | | + | |
| 8 | BCAL3190 | IclR family regulatory protein | | | + | |
| 9 | BCAM0466 | LysR family regulatory protein | | | + | |
| 10 | BCAM0466 | LysR family regulatory protein | | | + | |
| 11 | BCAM0618 | two-component regulatory system response regulator protein | | | + | |
| 12 | BCAM0820 | hybrid two-component system kinase-response regulator protein | | | + | |
| 13 | BCAM0886 | LysR family regulatory protein | | | + | |
| 14 | BCAM1415 | AraC family regulatory protein | | | + | |
| 15 | BCAM1755 | GntR family regulatory protein | | | + | |
| 16 | BCAM1975 | AraC family regulatory protein | | | + | |
| 17 | BCAS0235 | two-component regulatory system, response regulator protein | | | + | |
|  | **Membrane and transport** | | | | | |
| 1 | BCAL2005 | putative membrane protein | | | + | |
| 2 | BCAL2648 | putative outer membrane protein | | | + | |
| 3 | BCAL2648 | putative outer membrane protein | | | + | |
| 4 | BCAL2677 | putative permease protein | | | + | |
| 5 | BCAL2948 | putative membrane protein | | | + | |
| 6 | BCAL3490 | putative exported protein | | | + | |
| 7 | BCAM0837 | putative membrane protein | | | + | |
| 8 | BCAM0837 | putative membrane protein | | | + | |
| 9 | BCAM0945 | putative membrane protein | | | + | |
| 10 | BCAM1797 | putative ion channel protein | | | + | |
| 11 | BCAM2683 | putative cation-transporting ATPase membrane protein | | | + | |
|  | **Protein synthesis** | | | | | |
| 1 | BCAL0024 | GidA tRNA uridine 5-carboxymethylaminomethyl modification enzyme | | | + | |
| 2 | BCAL0115 | RpsU 30S ribosomal protein S21 | | | + | |
| 3 | BCAL0115 | RpsU 30S ribosomal protein S21 | | | + | |
| 4 | BCAL0423a | RpmH 50S ribosomal protein L34 | | | + | |
| 5 | BCAL0990 | RpmF 50S ribosomal protein L32 | | | + | |
| 6 | BCAL1553 | putative ribonuclease | | | + | |
| 7 | BCAL2880 | RpmF 50S ribosomal protein L32 | | | + | |
| 8 | BCAL3303 | queA S-adenosylmethionine:tRNA ribosyltransferase-isomerase | | | + | |
| 9 | BCAL3530 | HupA DNA-binding protein HU-alpha | | | + | |
| 10 | BCAM0131 | HchA chaperone protein | | | + | |
| 11 | BCAM0158 | putative diguanylate phosphodiesterase | | | + | |
|  | **Electron transport and ATP synthesis** | | | | | |
| 1 | BCAL0194 | putative oxidoreductase | | | + | |
| 2 | BCAL0389 | DsbC thiol:disulfide interchange protein | | | + | |
| 3 | BCAL0626 | putative 2-nitropropane dioxygenase | | | + | |
| 4 | BCAL3276 | PpnK NAD(+)/NADH kinase family protein | | | + | |
| 5 | BCAL3475 | putative molybdopterin-containing oxidoreductase | | | + | |
|  | **Chromosome replication** | | | | | |
| 1 | BCAL0423 | DnaA chromosomal replication initiation protein | | | + | |
| 2 | BCAL0423 | DnaA chromosomal replication initiation protein | | | + | |
|  | **Proteins involved in DNA methylation** | | | | | |
| 1 | BCAL0509 | MetK S-adenosylmethionine synthetase | | | + | |
|  | **Hypothetical proteins and pseudogenes** | | | | | |
| 1 | BCAL0362 | conserved hypothetical protein | | | + | |
| 2 | BCAL0610 | conserved hypothetical protein | | | + | |
| 3 | BCAL0761 | conserved hypothetical protein | | | + | |
| 4 | BCAL0763 | conserved exported protein | | | + | |
| 5 | BCAL0768 | conserved hypothetical protein | | | + | |
| 6 | BCAL0794 | conserved hypothetical protein | | | + | |
| 7 | BCAL0810 | pseudogene | | | + | |
| 8 | BCAL3259 | pseudogene | | | + | |
| 9 | BCAL3259 | pseudogene | | | - | |
| 10 | BCAL3483 | hypothetical protein | | | + | |
| 11 | BCAM0295 | conserved hypothetical protein | | | + | |
| 12 | BCAM0397 | conserved hypothetical protein | | | + | |
| 13 | BCAM0488 | conserved hypothetical protein | | | + | |
| 14 | BCAM0888 | conserved hypothetical protein | | | + | |
| 15 | BCAM2073 | hypothetical protein | | | + | |
| 16 | BCAM2254 | hypothetical protein | | | + | |
| 17 | BCAM2287 | hypothetical protein | | | + | |
| 18 | BCAM2417 | conserved hypothetical protein | | | + | |
| 19 | BCAM2609 | hypothetical protein | | | + | |
| 20 | BCAM2635 | hypothetical protein | | | + | |
| 21 | BCAS0773 | hypothetical protein | | | + | |
|  |  | **GTWWAC motif** | | |  | |
|  | Locus tag | Gene function | | Methylated in K56-2 | |  |
|  |  | **Intermediary metabolism** | |  | |  |
| 1 | BCAL0064 | AcoD acetaldehyde dehydrogenase | + | | |  |
| 2 | BCAL0162 | GmhA phosphoheptose isomerase | + | | |  |
| 3 | BCAL0508 | LpxL lipid A biosynthesis myristoyl acyltransferase | + | | |  |
| 4 | BCAL0709 | LipB lipoate-protein ligase B | + | | |  |
| 5 | BCAL0995 | AcpP acyl carrier protein | + | | |  |
| 6 | BCAL1290 | undecaprenyl pyrophosphate phosphatase | + | | |  |
| 7 | BCAL1467 | AroC chorismate synthase | + | | |  |
| 8 | BCAL1478 | putative hydrolase | + | | |  |
| 9 | BCAL1556 | RpiA ribose-5-phosphate isomerase A | + | | |  |
| 10 | BCAL1938 | family C40 cysteine peptidase | + | | |  |
| 11 | BCAL2406 | WabR putative glycosyltransferase | + | | |  |
| 12 | BCAL2419 | glycosyl hydrolases family protein | + | | |  |
| 13 | BCAL2701 | ArgD acetylornithine transaminase protein | + | | |  |
| 14 | BCAL2875 | AcpP acyl carrier protein | + | | |  |
| 15 | BCAL2942 | CysM cysteine synthase B | + | | |  |
| 16 | BCAL3153 | putative lipoprotein | + | | |  |
| 17 | BCAL3179 | LdhA putative D-lactate dehydrogenase | + | | |  |
| 18 | BCAM0013 | putative acetyltransferase | + | | |  |
| 19 | BCAM1679 | putative lysylphosphatidylglycerol synthetase | + | | |  |
| 20 | BCAM1761 | putative lipoprotein | + | | |  |
| 21 | BCAM2511 | GarD putative D-galactarate dehydratase | + | | |  |
| 22 | BCAM2737 | putative glycosyl transferase | + | | |  |
| 23 | BCAS0065 | putative glutathione S-transferase | + | | |  |
|  |  | **Membrane and transport** |  | | |  |
| 1 | BCAL0121 | AqpZ aquaporin Z | + | | |  |
| 2 | BCAL0126 | MotA chemotaxis protein | + | | |  |
| 3 | BCAL0824 | putative membrane protein | + | | |  |
| 4 | BCAL1525 | *flp* type pilus subunit | + | | |  |
| 5 | BCAL1808 | putative membrane protein | + | | |  |
| 6 | BCAL2301 | putative exported protein | + | | |  |
| 7 | BCAL2370 | putative membrane protein | + | | |  |
| 8 | BCAM0837 | putative membrane protein | + | | |  |
| 9 | BCAM0885 | putative membrane protein | + | | |  |
| 10 | BCAM0988 | putative exported protein | + | | |  |
| 11 | BCAM1669 | putative exported protein | + | | |  |
| 12 | BCAM1726 | putative exported protein | + | | |  |
| 13 | BCAM1743 | periplasmic solute-binding protein | + | | |  |
| 14 | BCAM1830 | putative exported protein | + | | |  |
| 15 | BCAM2555 | putative exported protein | + | | |  |
| 16 | BCAM2827 | putative exported protein | + | | |  |
| 17 | BCAM2828 | putative membrane protein | + | | |  |
|  |  | **Regulation** |  | | |  |
| 1 | BCAL0054 | MerR family regulatory protein | + | | |  |
| 2 | BCAL0444 | GntR family regulatory protein | + | | |  |
| 3 | BCAL0497 | two-component regulatory system, sensor kinase protein | + | | |  |
| 4 | BCAL2606 | two-component regulatory system, response regulator protein | + | | |  |
| 5 | BCAM0076 | TetR family regulatory protein | + | | |  |
| 6 | BCAM0085 | TetR family regulatory protein | + | | |  |
| 7 | BCAM0483 | ADA-like AraC family regulatory protein | + | | |  |
| 8 | BCAM2589 | IclR family regulatory protein | + | | |  |
| 9 | BCAS0007 | TetR family regulatory protein | + | | |  |
| 10 | BCAS0258 | GntR family regulatory protein | + | | |  |
|  |  | **Electron transport and ATP synthesis** |  | | |  |
| 1 | BCAL0080 | putative cytochrome | + | | |  |
| 2 | BCAL2145 | NADH-ubiquinone oxidoreductase subunit | + | | |  |
| 3 | BCAL2415 | PurT phosphoribosylglycinamide formyltransferase 2 | + | | |  |
| 4 | BCAL3187 | putative oxidoreductase | + | | |  |
| 5 | BCAL3362 | putative oxidoreductase | + | | |  |
| 6 | BCAL3432 | cytochrome c assembly protein | + | | |  |
|  |  | **Transposition** |  | | |  |
| 1 | BCAL2216 | putative transposase | + | | |  |
| 2 | BCAL3238 | putative transposase | + | | |  |
| 3 | BCAL3295 | putative transposase | + | | |  |
|  |  | **Protein synthesis** |  | | |  |
| 1 | BCAL1856 | RimO ribosomal protein S12 methylthiotransferase | + | | |  |
| 2 | BCALr3484 | tRNA-Val | + | | |  |
|  |  | **Chromosome replication** |  | | |  |
| 1 | BCAL0079 | *rep* ATP-dependent DNA helicase | + | | |  |
|  |  | **Proteins involved in DNA methylation** |  | | |  |
| 1 | BCAL0747 | putative methyltransferase | + | | |  |
|  |  | **Other proteins** |  | | |  |
| 1 | BCAL0825 | UvrA excinuclease ABC subunit A | + | | |  |
| 2 | BCAL0849 | subfamily M48B metalopeptidase | + | | |  |
| 3 | BCAL2643 | SodC superoxide dismutase | + | | |  |
| 4 | BCAL2749 | putative diguanylate phosphodiesterase | + | | |  |
| 5 | BCAL2940 | putative histone deacetylase-family protein | + | | |  |
| 6 | BCAM1362 | putative penicillin-binding protein | + | | |  |
| 7 | BCAS0010 | putative activator of osmoprotectant transporter | + | | |  |
| 8 | BCAS0293 | AidA nematocidal protein | + | | |  |
|  |  | **Hypothetical proteins and pseudogenes** |  | | |  |
| 1 | BCAL0362 | conserved hypothetical protein | + | | |  |
| 2 | BCAL0434 | putative exported protein | + | | |  |
| 3 | BCAL1442 | conserved hypothetical protein | + | | |  |
| 4 | BCAL2175 | conserved hypothetical protein | + | | |  |
| 5 | BCAL3193 | conserved hypothetical protein | + | | |  |
| 6 | BCAM0008 | conserved hypothetical protein | + | | |  |
| 7 | BCAM1874-2 | pseudo | + | | |  |
| 8 | BCAM2207 | conserved hypothetical protein | + | | |  |
| 9 | BCAM2289 | conserved hypothetical protein | + | | |  |
