# Supplementary material for: DNA Methylation Epigenetically Regulates Gene Expression in Burkholderia cenocepacia and Controls Biofilm Formation, Cell Aggregation, and Motility
Source: mSphere. 2020 Jul 15;5(4):e00455-20. doi: 10.1128/mSphere.00455-20 (PMC7364216; doi:10.1128/mSphere.00455-20)
Supplement: TABLE S3 [file mSphere.00455-20-st003.docx]

| **Transcription Factor** | **Strand** | **Score** | **Consensus sequence** |
| --- | --- | --- | --- |
| **CACAG motif** | | | |
| **GlpR** | - | 6.07 | **TGTG**TTCTAATTTCATTTAG |
| **GTWWAC motif** | | | |
| **ArcA** | + | 7.39 | T**GTTAAC**ATG |
| **ArcA** | - | 7.22 | T**GTTAAC**ACG |
| **OxyR** | - | 4.49 | CAT**GTTAAC**AC |
| **OxyR** | + | 4.11 | CGT**GTTAAC**AT |
| **Fis** | - | 3.49 | GACGCGCAT**GTTAAC** |
| **Fur** | - | 2.55 | AT**GTTAAC** |
