# Supplementary material for: DNA Methylation Epigenetically Regulates Gene Expression in Burkholderia cenocepacia and Controls Biofilm Formation, Cell Aggregation, and Motility
Source: mSphere. 2020 Jul 15;5(4):e00455-20. doi: 10.1128/mSphere.00455-20 (PMC7364216; doi:10.1128/mSphere.00455-20)
Supplement: TABLE S4 [file mSphere.00455-20-st004.docx]

| Primers | | Sequence (5' 3') | | | | Abbreviation | | |
| --- | --- | --- | --- | --- | --- | --- | --- | --- |
| ΔBCAL3494 primers | | | | | | | | |
| Flanking sequences | |  | | |  | | | |
| upstream sequence F | | ATATGAATTCCCAACGGTTTCAAGGAGACG | | | UL3494-EcoRI | | | |
| upstream sequence R | | ATATAGATCTGGCGGATCGATGTAGACGAG | | | UL3494-Bgl II | | | |
| downstream sequence F | | ATATAGATCTGGGATGCAAGAAGGCTCATC | | | DL3494-Bgl II | | | |
| downstream sequence R | | TTTACCCGGGATAGGTCTCGCGCTGGTGTC | | | DL3494-SmaI | | | |
| Control primers | |  | | |  | | | |
| Overlapping sequence insert control F | | ATGGAGAATCCCGGAAGAAG | | | joinL3494-F | | | |
| Overlapping sequence insert control R | | TGCTGTTTCATCTGGTGCTC | | | joinL3494-R | | | |
| BCAL3494 gene control F | | GGCAGCGATTTCGTCTATCC | | | geneL3494-F | | | |
| BCAL3494 gene control R | | CACTTCGTGCTCGTCGATGT | | | geneL3494-R | | | |
| Complementation | |  | | |  | | | |
| BCAL3494 complementation F | | TTTGGATCCTCGTCCTGTTTCAGCCTTTGAGC | | | L3493-4-ov-BamHI | | | |
| BCAL3494 complementation R | | TTTTCTAGAGCTTTCACGCGAATGACAGGATG | | | L3493-4-ov-XbaI | | | |
| ΔBCAM0992 primers | | | | | | | | |
| Flanking sequences | |  | | |  | | | |
| upstream sequence F | | ATATGAATTCGATCTACCTGAAGCGCGAAG | | | UM0992-EcoRI | | | |
| upstream sequence R | | ATATGCTAGCGGCTCTTCGATCAGGTCACG | | | UM0992-NheI | | | |
| downstream sequence F | | ATATGCTAGCCGTATGAGACCGGAGCAAGC | | | DM0992-NheI | | | |
| downstream sequence R | | ATATAGATCTCACTTGACCCACAGGCCTTC | | | DM0992-Bgl II | | | |
| Control primers | |  | | |  | | | |
| Overlapping sequence insert control F | | ATACCTCGGTGCAGCTGATC | | | joinM0992-F | | | |
| Overlapping sequence insert control R | | CAATGCTCGAAACATCCAGA | | | joinM0992-R | | | |
| BCAM0992 gene control F | | AACGATTCGGACAAGCGTTC | | | geneM0992-F | | | |
| BCAM0992 gene control R | | CGGTCCCAGATGATCTCGTT | | | geneM0992-R | | | |
| Complementation | |  | | |  | | | |
| BCAM0992 complementation F | | AATAATAATCATATGCGTGACCTGATCGAAGAG | | | M0992-ov-NdeI | | | |
| BCAM0992 complementation R | | TTTGGATCCCATACGATGTATGCGTTGCGTTC | | | M0992-ov-BamHI | | | |
| pGPI-SceI-XCm MCS primers | | | | | | | | |
| MCS plasmid sequence insert control F | | AACAAGCCAGGGATGTAACG | | | MCS-B-F | | | |
| MCS plasmid sequence insert control R | | TGTTCGGCCAGATAGAAACC | | | MCS-B-R | | | |
| qPCR primers | |  | | |  | | | |
| Primers | | Sequence (5' 3') |  |  | | |  |  |
| M0918-F | | GAGATGAGCACCGATCACAC |  |  | | |  |  |
| M0918-R | | CCTTCGAGGAACGACTTCAG |  |  | | |  |  |
| L0003-F | | AATGGCCTGAATTCCTGACG |  |  | | |  |  |
| L0003-R | | GTGATGCACGGTCTTCTTCG |  |  | | |  |  |
| L0423-F | | AGCTGGACTGGGTCAAGAGC |  |  | | |  |  |
| L0423-R | | GGATCGAGGACGAACTGGAC |  |  | | |  |  |
| M0820-F | | ACGTCTACCGGACCGAACAC |  |  | | |  |  |
| M0820-R | | TCGAGCACGATTTCGTTGAG |  |  | | |  |  |
| L1059-F | | GATGCTGACGACGAACGAAC |  |  | | |  |  |
| L1059-R | | GTCCTTGAAGATGCCGAAGC |  |  | | |  |  |
| L1457-F | | CAGCAGATGAATTCGACCAC |  |  | | |  |  |
| L1457-R | | TCGACGTAAGCGAGGATCTG |  |  | | |  |  |
| S0223-F | | ATGCTCGTGTCGTTTCATGC |  |  | | |  |  |
| S0223-R | | ACTGGTCGCCGTAGTCGAAG |  |  | | |  |  |
| M2738-F | | GCTGAGCGAACAGGTTGACG |  |  | | |  |  |
| M2738-R | | ACCATTCGGCCTTCACTTCC |  |  | | |  |  |
| M1262-F | | ACCGCGAACTCGATGAACTG |  |  | | |  |  |
| M1262-R | | GGTGCAGGATCGTGTTGGTC |  |  | | |  |  |
| L0079-F | | GTCAACCAGCTCACCGTCTG |  |  | | |  |  |
| L0079-R | | CTTCCACAGCGAGATGATGC |  |  | | |  |  |
| L0126-F | | TGATGGCGCTTCTTTACGTG |  |  | | |  |  |
| L0126-R | | ATTCGACGATGTGGTGATCG |  |  | | |  |  |
| L0508-F | | TCGTCTGAGGGTGTTCAAGC |  |  | | |  |  |
| L0508-R | | ATCAGCGGAATCTGCTCCTC |  |  | | |  |  |
| L0709-F | | GCGGCGTATAATCTCGCTTC |  |  | | |  |  |
| L0709-R | | ATGTCGACGGTTTCCAGTCC |  |  | | |  |  |
| L2701-F | | TCACGTTCGACCACAGCTTC |  |  | | |  |  |
| L2701-R | | GACGCGATGTTGTTCAGCTC |  |  | | |  |  |
| L2942-F | | AAGCCTACATGCCGACCATC |  |  | | |  |  |
| L2942-R | | GATCGCAGACGATGAACACG |  |  | | |  |  |
| L2406-F | | TGCCGAGATTGCTGTTCAAG |  |  | | |  |  |
| L2406-R | | AGCAACGGTGTCAGCAACAG |  |  | | |  |  |
| L0024-F | | TACAGGCGTGATCGAAGGTG |  |  | | |  |  |
| L0024-R | | GGAAGATCTGGTGCGATTCC |  |  | | |  |  |
| L0509-F | | GGTGATGGTCAACACGTTCG |  |  | | |  |  |
| L0509-R | | CCGTAAGCTGCCGTCTTCTC |  |  | | |  |  |
| L2465-F | | GGCTGTCTGATCGTGCTGTC |  |  | | |  |  |
| L2465-R | | ATGCCCTGTTGAACCGTCAC |  |  | | |  |  |
| L2767-F | | AGACCTATCACCCGCTGCAC |  |  | | |  |  |
| L2767-R | | ACGGGTGGTATTCGTTCGTC |  |  | | |  |  |
| L2782-F | | AGCCGAACACGATGACACTC |  |  | | |  |  |
| L2782-R | | CTTGCGGCTTTCGTAATTGG |  |  | | |  |  |
| L3303-F | | GACGAGACGCGCTACCAGAC |  |  | | |  |  |
| L3303-R | | GGTCGTACCACTCGCTGTGC |  |  | | |  |  |
| L0054-F | | AGCGCACCGATTCGAACTAC |  |  | | |  |  |
| L0054-R | | ACGTGTCCGATGTGATCGTC |  |  | | |  |  |
| L0162-F | | GCATCCACGAAGTCCATCTG |  |  | | |  |  |
| L0162-R | | AATCCTCACCCAGCAGCATC |  |  | | |  |  |
| L1556-F | | ACTGCTTCATCGACGCACTC |  |  | | |  |  |
| L1556-R | | CGTTCAGGTCGAACACCTTG |  |  | | |  |  |
| L2415-F | | GTGAAGCCCGTGATGTCGTC |  |  | | |  |  |
| L2415-R | | GACCGGCTCGCAGAAGTAGG |  |  | | |  |  |
| S0258-F | | AAGATGCGGGAACTGATCGAC |  |  | | |  |  |
| S0258-R | | ATGAAACACCCAGCCGATACG |  |  | | |  |  |
| M0941-F | | ACAAGCAATCGGTGTGATCG |  |  | | |  |  |
| M0941-R | | AGCGTATAGGTCGGCACCAG |  |  | | |  |  |
| M1415-F | | AGACGACAACGCGAAACTCG |  |  | | |  |  |
| M1415-R | | ATCAGGTACGACGGCGACAG |  |  | | |  |  |
| M0076-F | | TGCCGCCTTTGTACTCATGG |  |  | | |  |  |
| M0076-R | | GCGACACGGAAATGATCTCG |  |  | | |  |  |
| M1362-F | | GATCGTGGTCGTCGTGTTCC |  |  | | |  |  |
| M1362-R | | GTCTTGTCGTTGCCGAGACG |  |  | | |  |  |
| L1515-F | | CGCAAGCAACCTGTACTTCG |  |  | | |  |  |
| L1515-R | | GTCAGGCGATTCAGGATGTG |  |  | | |  |  |
| eGFP primers |  |  | | | | | |  |
| BCAL0079 Forward |  | ATATGGATCCTGCGTATTGTGTCCGATCA | | | | | |  |
| BCAL0079 Reverse |  | ATATGAATTCCATGATGGCGGATGGTGTT | | | | | |  |
| BCAL1515 Forward |  | ATATGGATCCGGTGCTTTCAGGCACATTTC | | | | | |  |
| BCAL1515 Reverse |  | ATATGAATTCGCCGAACAGATAGGAGTTCAG | | | | | |  |
| BCAM0820 Forward |  | ATATGGATCCCTGCCGATTCGGAGTATCTG | | | | | |  |
| BCAM0820 Reverse |  | ATATGAATTCATCCGAGGCATTATCACTGCT | | | | | |  |
| Insert plasmid Forward |  | CGTAGAGGATCTGCTCATGTTTGAC | | | | | |  |
| Insert plasmid Reverse |  | GACGTAAACGGCCACAAGTTCA | | | | | |  |
